# T cell subtype profiling measures exhaustion and predicts anti-PD-1 response

**DOI:** 10.1038/s41598-022-05474-7

**Published:** 2022-01-25

**Authors:** Ian Schillebeeckx, Jon Earls, Kevin C. Flanagan, Jeffrey Hiken, Alex Bode, Jon R. Armstrong, David N. Messina, Douglas Adkins, Jessica Ley, Ilaria Alborelli, Philip Jermann, Jarret I. Glasscock

**Affiliations:** 1 Cofactor Genomics, Inc., San Francisco, CA USA; 2 Washington University School of Medicine, Saint Louis, MO USA; 3 Department of Medical Genetics and Pathology, University Hospital Basel, Basel, Switzerland

**Keywords:** Adaptive immunity, Computational models, Immunotherapy

## Abstract

Anti-PD-1 therapy can provide long, durable benefit to a fraction of patients. The on-label PD-L1 test, however, does not accurately predict response. To build a better biomarker, we created a method called T Cell Subtype Profiling (TCSP) that characterizes the abundance of T cell subtypes (TCSs) in FFPE specimens using five RNA models. These TCS RNA models are created using functional methods, and robustly discriminate between naïve, activated, exhausted, effector memory, and central memory TCSs, without the reliance on non-specific, classical markers. TCSP is analytically valid and corroborates associations between TCSs and clinical outcomes. Multianalyte biomarkers based on TCS estimates predicted response to anti-PD-1 therapy in three different cancers and outperformed the indicated PD-L1 test, as well as Tumor Mutational Burden. Given the utility of TCSP, we investigated the abundance of TCSs in TCGA cancers and created a portal to enable researchers to discover other TCSP-based biomarkers.

## Introduction

Anti-PD-1 therapies are an increasingly important treatment option across many cancer types^1,2^. Anti-PD-1 therapy and other checkpoint inhibitors are approved for the treatment of 14 cancer types, making around 39%^3^ of all cancer patients eligible for checkpoint therapy. Unfortunately, only approximately 11%^3^ of all cancer patients benefit from anti-PD-1 therapies. Yet, among patients who respond to anti-PD-1 therapy, many experience a robust, durable response even in cancers with historically poor long-term survival^4,5^. In an effort to unlock these improved outcomes and more cost-efficient treatments, researchers have investigated various biomarkers^6^. The most commonly used biomarker for anti-PD-1 therapies is PD-L1 expression measured by IHC. PD-L1 is the ligand for the PD-1 receptor and a target of checkpoint inhibitors in its own right. Unfortunately, PD-L1 is an unreliable biomarker for predicting response^7^.

Although the PD-L1 molecule is involved in the mechanism of action of anti-PD-1 therapies, other aspects of adaptive immunity, namely T cells, may be more useful in predicting response^8^. T cells can be classified according to their activation and differentiation status, which capture the activity, antigen-exposure, and specific functional role of a T cell population. In particular, five T cell subtypes (TCSs)—naïve^9^, activated^10^, effector memory (EM)^11^, central memory (CM)^11^, and exhausted^12^—are descriptive of the immunogenic status of T cell adaptive immune response and, when measured in isolation, have offered insights into response to anti-PD-1 therapy. For instance, in Head and Neck Squamous Cell Carcinoma (HNSCC), higher levels of EM T cells are associated with response^13^, while exhausted T cells are associated with poor prognosis^13,14^. PD-1 inhibits effector function upon ligand binding and is heavily expressed in exhausted T cells^12^, and has been associated with response in Non-Small Cell Lung Cancer (NSCLC)^15,16^. In addition, exhausted T Cells are an important component of the anti-tumor immune response following PD-1 or CTLA-4 blockade in several cancers^17–23^. Furthermore, higher levels of CM T cells have been associated with anti-PD-1 response in melanoma^24^. These works suggest that a more nuanced and comprehensive characterization of TCSs might more successfully predict anti-PD-1 response.

With this goal, we developed a biomarker platform that estimates the prevalence of these five TCSs using novel RNA models, called Subtype Health Expression Models (sHEMs). This novel approach has several advantages: 1) Estimating TCSs using sHEMs is easy to perform on commonly available clinical samples. Traditional methods of measuring TCSs, especially the activated and exhausted subtypes, require functional assays and/or flow cytometry and are difficult or impossible to perform routinely on common clinical specimens. Similar to Immune Health Expression Models^25^, sHEMs eliminate this limitation and allow characterization of TCSs in formalin fixed and paraffin embedded (FFPE) tissues. 2) The sHEMs do not rely on canonical single analyte measurements (like PD-1) and thus more specifically characterize TCSs. 3) Substantial RNA-seq datasets are available for validating and applying RNA-based sHEMs. This RNA-based biomarker approach mirrors other commercial tests like Oncotype DX^26^, Veracyte Afirma^27^, and Agendia MammaPrint^28^, which have found clinical utility in other oncology indications.

Other work has looked at using gene signatures to measure the immune component of tumors and predicting response to anti-PD-1 therapy. Digital cytometry or cell deconvolution methods enable researchers to estimate the abundance of certain cell types using RNAseq data. Methods such as ESTIMATE^29^, Cibersort^30^, CibersortX^31^, quanTIseq^32^, xCell^33^, MCP-counter^34^, and EPIC^35^ are generally derived from public datasets including microarray and RNAseq data. These methods quantify the abundance of lymphocytes at a high level, i.e. CD4+ and CD8+ , and not at a more granular functional level, preventing the full characterization of all important TCSs. XCell enables the estimation of enrichment of naïve, EM, and CM TCSs for both CD4+ and CD8+ , but lacks activated and exhaustion states. Gene expression of T cell infiltration has been previously considered for predicting response to anti-PD-1 therapy. Inflammation^36^, cytotoxicity^37^, IFNγ^38,39^, antigen presentation^39^, and exhaustion^39^ gene signatures, have been explored, but have yet to be adopted into oncology practice.

In this work, we use sHEMs to predict response to anti-PD-1 therapy in three indications. First, we show how we built and used sHEMs to characterize the five TCSs. Then, we tested that the process of characterizing TCS using these sHEMs, called T Cell Subtype Profiling (TCSP), is analytically robust. Next, we validated TCSP in CD39+ and PD-1+ T cell isolates from tumors and show that CD39 and PD-1 surface markers are not specific to exhaustion. In addition, we characterized how virus infection can affect the TCS breakdown of T cell infiltrate in cancers with viral etiologies. Then, we used TCSP to characterize HNSCC, NSCLC, and Melanoma cohorts and show multiple associations between TCSs and response to anti-PD-1 therapy. Leveraging these insights, we built multianalyte biomarkers in these three indications using machine learning. These TCSP-based biomarkers predicted response to anti-PD-1 therapies in HNSCC, NSCLC, and Melanoma, and out-performed the on label PD-L1 test and Tumor Mutational Burden (TMB). Finally, we used TCSP to explore other cancer types in The Cancer Genome Atlas (TCGA). Given its robustness and practicality, we have made the TCSP platform available (via tcsp.cofactorgenomics.com) to other researchers so that they may characterize TCSs in their own datasets and investigate other biomarkers.

## Results

### Subtype health expression models discriminate T cell subtypes

**Figure 1:**
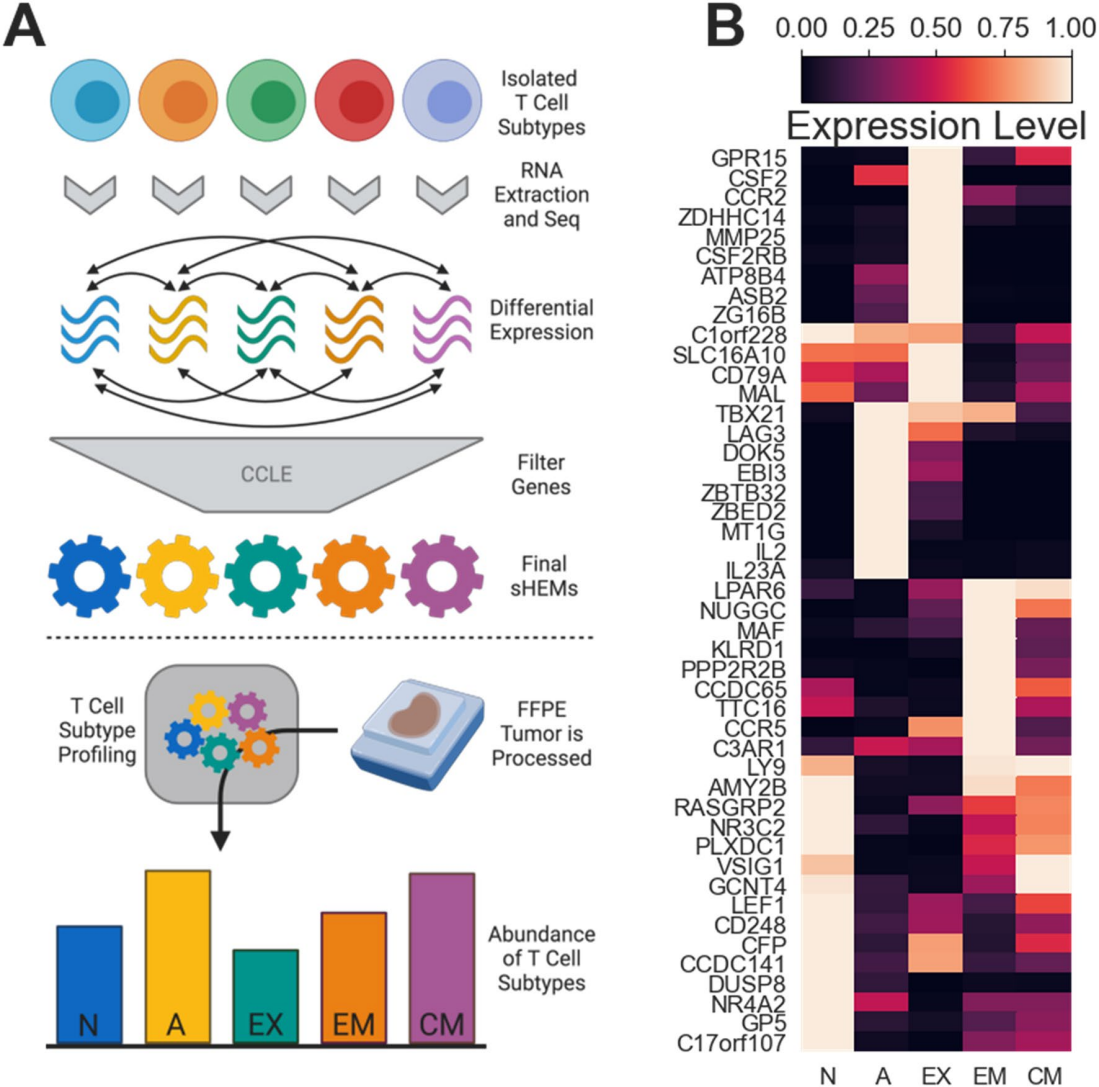
Subtype Health Expression Models discriminate T cell subtypes. (**A**) Schematic of the approach to creating data and using data-driven Subtype Health Expression Models (sHEMs). (**B**) A heatmap shows the gene-normalized expression of genes that comprise the 5 sHEMs. *N* naïve model; *A* activated model; *EX* exhausted model; *EM* effector memory model; *CM* central memory model.

We created sHEMs as a tool to estimate the abundance of TCSs in newly sequenced FFPE samples or in public datasets. To create them, we isolated cells that define each TCS (Fig. 1A), including naïve, effector memory (EM), and central memory (CM) CD8+ T cells, from healthy PBMC donors using flow cytometry. Activated and exhausted T cells were generated in vitro via continuous CD3/CD28/CD2 stimulation of isolated naïve CD8+ T cells. Activated T Cells corresponded to early stimulation and had maximal proliferative capacity according to IL2 and IFNγ expression. In contrast, exhausted T cells corresponded to late chronic stimulation having impaired cytokine production, little to no proliferation, and high expression of PD-1, TIM3, and LAG3 inhibitory receptors, in agreement with observations in literature^12^.

Total RNA isolated from these five types of cell isolates were respectively processed and sequenced. Differentially expressed genes were chosen to identify the five TCSs (Fig. 1A). sHEMs for each of the five TCSs were created using the mean value for each of the differentially expressed genes for the five respective isolates. This data-driven approach has an advantage of being unbiased, which is especially important given the overlap between classical effector and exhaustion genes. These initial models adequately estimated TCS abundance in simple, PBMC based samples, but suffered from high false positive estimates in more heterogeneous tumor samples. To improve performance in complex samples, genes with relatively high expression in the cell lines of the Cancer Cell Line Encyclopedia (CCLE)^40^ were filtered out (Fig. 1A). The resulting five sHEMs were composed each of 46 genes (Fig. 1B).

The five sHEMs differentiate the five TCSs in heterogeneous FFPE tumor samples. Using these models and gene expression data from a sample, an unknown FFPE tumor sample can be characterized by solving a linear equation. The estimated abundances of each of the five TCSs represent what ratio of RNA in a whole sample is comprised of each TCS (Fig. 1A) and are reported as a number between 0 and 100. Often, for example when comparing against other measurement modalities or previous work, it is most useful to consider TCS estimates normalized to their sum. In this case, axis labels are reported as “TCS $\overline{{{\text{Estimate}}}}$” and are reported as a number between 0 and 1. We refer to the process of characterizing a sample in regard to TCSs as T Cell Subtype Profiling (TCSP). TCSP characterizes the immune response in a tumor and, as shown later in this work, can predict patient response to anti-PD-1 therapy. sHEMs were created using whole exome sequencing data, enabling TCSP to characterize the infiltrating immune response within and across many cancer types using publicly available RNA-seq data.

We show the normalized expression of each gene across all five sHEMs (Fig. 1B, Supplementary Fig. 1). Per the Reactome database^41^, effectively all of these genes are involved in immunity related pathways (Supplementary Fig. 2). Each model had a few notable, constituent genes which characterize each TCS. The naïve sHEM displayed high expression of LEF1, a gene involved in T cell development and peripheral T cell differentiation^42^. A set of genes involved in homeostasis (NR4A2^43^) and quiescence (CD248^44^ and DUSP8^45^) were also highly expressed in the naïve model versus others. The activated sHEM showed high expression of cytokines related to inflammation and proliferation including EBI3^46^, IL2^47^, and IL23A^48^. The transcription factor TBX21 (T-Bet), which is involved in the regulation of development and CD4+ T Cell differentiation^49^, was also highly expressed. The inhibitory receptor, LAG3 (HAVCR2), negatively regulates T cell activation^50,51^ and was also a constituent gene of the activated sHEM. The exhausted sHEM had high expression of CSF2, a gene associated with prolonged stimulation and cell aging^52^. This model also had the highest expression of genes that prohibit differentiation (ASB2^53^), cell growth (CSF2RB^52^), and inflammation (CCR2^54^). The EM sHEM had high expression of KLRD1 (CD94), which may regulate effector functions and cell survival of CD8+ T cells^55^. Additionally, MAF, a regulator of differentiation and function in a wide variety of T cells^56^, and CCR5, a gene involved in chemokine-induced costimulation^57^, were highly expressed. The CM sHEM had high expression of LY9, a gene that negatively regulates the development of memory CD8+ T cells^58^. In addition, genes associated with trafficking (GCNT4^59^) and adhesion (VSIG1) were also more highly expressed in the CM model.

Interestingly, some canonical genes associated with the five TCSs in literature are missing from the sHEMs due to our data-driven approach. These genes are not differentially expressed between the five TCSs nor between the five TCSs and the tumor microenvironment (via the CCLE database) and therefore aren’t useful for estimating abundances in the tumor microenvironment. For example, the exhausted sHEM does not include other inhibitory receptors such as PD-1 (PDCD1) and TIM3 (HAVCR2)^12^ because these genes are also highly expressed in the activated subtype. Genes such as TCF7, TOX, EOMES, and CD39 (ENTPD1)^12^ were also not discriminative (Supplementary Fig. 1 and 3).

### T cell subtype profiling is analytically robust

The five sHEMs were developed with the goal to characterize the immune response in heterogeneous specimens and predict response to anti-PD-1 therapy. Therefore, it is imperative that TCSP is accurate and analytically robust. This section focuses on the analytical experimentation done to validate the five sHEMs and specifically, the abundances estimated by the TCSP technique.

**Figure 2:**
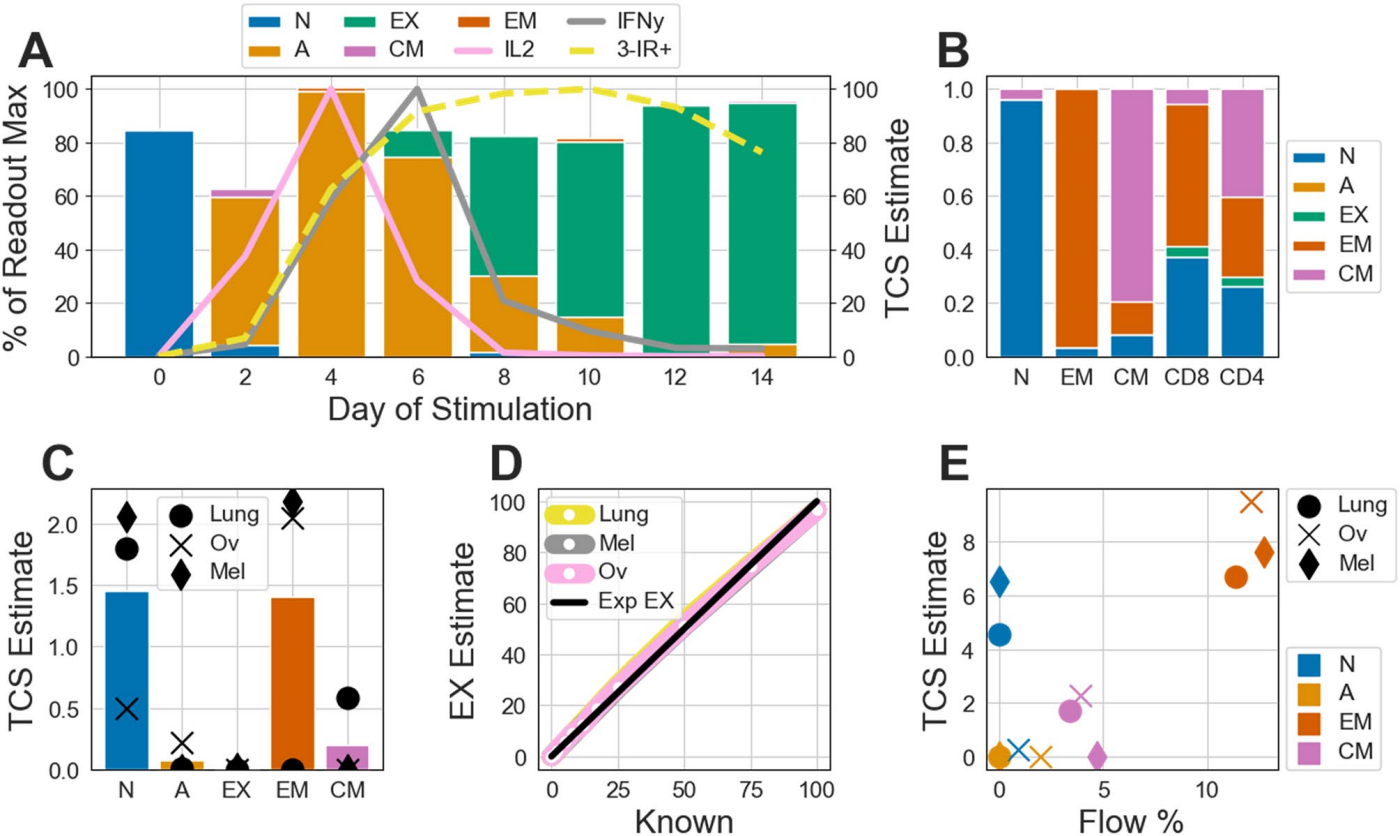
T Cell Subtype Profiling is analytically robust. (**A**) The transient change of naïve CD8+ T cells during a chronic stimulation in vitro model. Naïve CD8+ T cells were isolated from a donor and stimulated for 14 days. This donor was not used in creating the Subtype Health Expression Models (sHEMs). Every two days, the presence of the extracellular cytokines IL2 and IFNγ was measured via ELISA and the abundance of cells jointly expressing the PD1, TIM3, and LAG3 inhibitory receptors (3-IR+) was measured via flow cytometry. The IL2, IFNγ, and 3-IR+ readouts are independently normalized to their max expression during the time series and shown as lines (left y-axis). Cells were harvested every two days and characterized using T Cell Subtype Profiling (TCSP). T Cell subtype (TCS) estimates are shown as stacked bars (right y-axis). *N* naïve subtype; *A* activated subtype; *EX* exhausted subtype; *EM* effector memory subtype; *CM* central memory subtype. (**B**) The sum-normalized TCS estimates of T Cell populations isolated from blood: naïve CD8+ T cells (N, n = 5), effector memory CD8+ T Cells (EM, n = 5), central memory CD8+ T Cells (CM, n = 3), CD8+ T cells (CD8, n = 8), and CD4+ T cells (CD4, n = 8). (**C**) TCSP of CD45− cells isolated from lung adenocarcinoma (Lung, n = 1), ovarian adenocarcinoma (Ov, n = 1), and melanoma (Mel, n = 1) tumor samples. Numbers shown are on a scale of 0 to 100. Bars show average estimated abundance of each TCS across all three tumors (points). (**D**) Exhaustion subtype estimates of synthetic samples comprised of varying fractions of chronically stimulated CD8+ T cells (day 14 from **A**) and CD45− cells isolated from tumor samples (from **C**). The expected exhaustion estimates (Exp EX) are shown as a black line. (**E**) TCSP of dissociated whole tumor samples (from **C**) compared against % of CD45+ immune cells determined by canonical flow cytometry estimation (Flow %). Point shapes correspond to cancer type, while colors correspond to TCS.

First, we demonstrate the performance of estimating the abundance of the naïve, activated, and exhausted TCSs. Naïve CD8+ cells from a donor withheld from creating the models were chronically stimulated in vitro for 14 days. At days 4 and 6, there is a peak abundance of extracellular IL2 and IFNγ cytokines, respectively (Fig. 2A). Meanwhile, the abundance of cells triple-positive for PD-1, TIM3, and LAG3 receptors grow through day 4 and peak at day 10, with sustained abundance into days 12 and 14 (Fig. 2A, Supplementary Fig. 4B). In accordance with previous studies^12,60–62^, there is also: a progressive increase in expression of TIGIT, 2B4, CD39 (ENTPD1), and TOX; a progressive decrease in expression of LAG3 and GZMB after peak activation; and a peak of EOMES expression early and at the end of chronic stimulation (Supplementary Fig. 4). This progressive increase in several inhibitory receptors, coupled with progressive loss of proliferative and cytotoxic expression and a later stabilization or decrease of inhibitory receptors is a hallmark of T cell exhaustion^12^. These readouts suggest that in this chronic stimulation experiment the cells start out as naïve, became activated by day 4 and are exhausted by days 12 and 14. We compared these orthogonal measurements to TCS estimates. Our characterization mirrored this trend as the cells on day 0 are estimated to be naïve, day 4 is chiefly characterized to be activated, days 6 through 10 are characterized as a progressive transition from a population of activated to exhausted, while days 12 and 14 are estimated to be exhausted (Fig. 2A). These trends are preserved regardless of which donors are used for creating the sHEMs and which withheld donor is used for evaluation (Supplementary Fig. 5). The xCell method observes a state switch on Day 2 from Naïve, but lacks any ability for determining the activation or exhaustion of the CD8+ cells, instead suggesting that late (days 10–14) stimulated cells are enriched for EM (Supplementary Fig. 6A).

Increased inhibitory receptor levels alone are not sufficient to specifically measure the exhaustion of T cells (Fig. 2A). Rather, it is the measurement of secreted cytokine levels after stimulation coupled with inhibitory receptor expression that enables one to approximate exhaustion in of a population of T cells. Performing this sort of multi-faceted analysis is not only laborious, but also infeasible in FFPE tumor samples. Our exhausted sHEM addresses these challenges and thus provides a powerful tool in profiling dysfunction of the immune response in a preserved tumor. We next evaluated the performance of TCSP using PBMCs. For a single donor, live T cells were sorted for naïve, EM, and CM CD8+ cells via flow cytometry. We did not sort for activated or exhausted T cells, which are typically rare in healthy patient PBMCs and are difficult to accurately characterize with flow markers alone. Samples were profiled and normalized to the total estimated abundance of all five TCSs. TCSP correctly characterized the naïve and EM isolates as predominately being naïve and EM subtypes, respectively. The CM isolate was estimated to be ~ 80% the CM subtype, but also estimated some fraction of the isolate to be naïve and EM subtypes (Fig. 2B). We also profiled the CD4+ and CD8+ isolates of PBMCs from eight donors. The CD8+ isolates had a mean estimate of 37% naive, 0% activated, 4% exhausted, 53% EM, and 5% CM, while the CD4+ isolates had a mean estimate of 26% naïve, 0% activated, 4% exhausted, 30% EM, and 40% CM. The estimated abundances of the naïve, EM, and CM TCSs in CD4+ and CD8+ T cells reflect those reported in other healthy donors^63^. The CD4+ and CD8+ samples are estimated to have a low level of exhaustion, perhaps due to latent viral infections, for example from Epstein-Barr Virus, where up to 2.5% of CD8+ T cells are specific to EBV in healthy individuals^62,64^. In general, the xCell method struggles with these T cell isolates, exhibiting false positives of high CD4+ cell enrichment across all isolates and an inability to differentiate between EM and CM isolates (Supplementary Fig. 6B). These results suggest that TCSP can accurately estimate TCSs across both CD4+ and CD8+ T cells and is superior to the xCell T cell enrichment method.

TCSP of the tumor microenvironment is challenging because immune cells are integrated in and affected by a heterogenous mix of tumor and stroma. Therefore, we next aimed to validate performance with various isolates of dissociated tumor cells from single donors of lung cancer, melanoma, and ovarian cancer. CD45− isolates are devoid of immune cells and were sorted to establish the specificity of TCS estimates. The average estimates across the three tissue types are < 0.25 (out of 100 parts) for the activated and CM subtypes, and effectively 0 for the exhausted subtype (Fig. 2C). On average, the naïve and EM subtypes suffered from higher false positive estimation, although still very low and to different degrees depending on the cancer type (Fig. 2C). We further sought to explore the sensitivity of profiling the exhausted subtype by titrating RNA-seq data from an exhausted sample (from day 12 of the chronic stimulation) into the CD45− sample, in silico. In fractions of 1% to 100% exhausted reads, we see a reliable estimate when independently titrating in the 3 different cancer types (Fig. 2D). In these titrations, the level of the other four TCSs are at or near 0 (Supplementary Fig. 7) as expected. Finally, we measured the unsorted lung, melanoma, and ovarian samples, which consisted of a mix of immune, stromal, and cancer cells. Estimates of these three samples correlate with flow cytometry measurements for EM, CM, and activated TCSs (Fig. 2E). The naïve subtype was estimated to be more abundant than what was measured by flow cytometry, which may be a result of reduced specificity for this TCS (Fig. 2C). As described before, it is not possible to functionally characterize exhaustion level with flow cytometry alone, thus we were not able to evaluate our exhaustion estimates in these 3 samples. However, when comparing CD45+ isolates to the unsorted samples, the rank order of exhausted subtype estimates is preserved among all TCSs (Supplementary Fig. 8). In all, these results build confidence in TCSP of infiltrating T cells in heterogeneous tumor samples.

### T cell subtype profiling is consistent with external observations

TCSP is robust in estimating the abundance of TCSs and can be used to characterize public datasets, making TCSP unique in its ability to investigate many biological and clinical questions across specimen types and datasets. In addition, the breadth of our functionally validated sHEMs enables a detailed, yet comprehensive approach to investigate TCSs in the context of chronic infection and cancer. As such, we next sought to validate our approach by corroborating external observations in literature.

**Figure 3:**
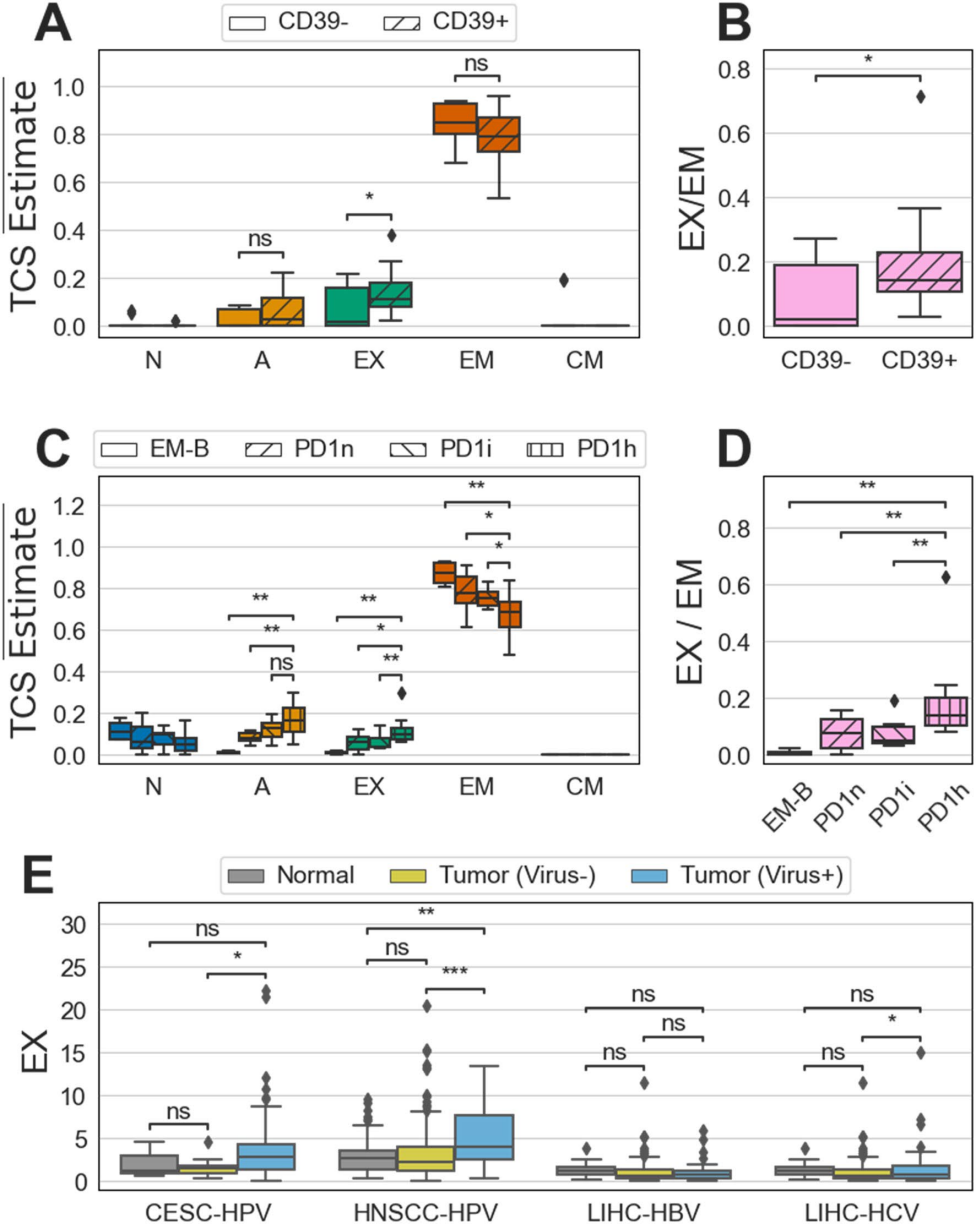
T Cell Subtype Profiling is consistent with external observations. (**A**) The sum-normalized T Cell subtype (TCS) estimates (TCS $\overline{{{\text{Estimate}}}}$) for CD39− (n = 11) and CD39+ (n = 12) CD8+ T cells isolated from Non-small Cell Lung Cancer (NSCLC) and Colorectal Cancer (CRC) tumors. The distribution of estimates per group is shown as a box and whisker. The box represents the 1st and 3rd quartiles, while the center line represents the median. The whiskers encompass 1.5 times past the 1st and 3rd interquartiles and points outside this range are shown as diamonds. *N* naïve subtype; *A* activated subtype; *EX* exhausted subtype; *EM* effector memory subtype; *CM* central memory subtype. (**B**) A composite exhaustion level for CD39− and CD39+ isolates. The composite level is calculated as exhausted divided by EM (**C**) The sum-normalized TCS estimates for CD8+ T cell isolates sorted by varying levels of PD1 expression as measured by flow cytometry. EM CD8+ T cells isolated from blood (EM-B, n = 4) were compared against CD8+ T cells isolated from NSCLC tumors with no PD1 (PD1n, n = 11), intermediate PD1 (PD1i, n = 11), and high PD1 (PD1h, n = 11) expression. (**D**) The composite exhaustion level for the same isolates as (**C**). The composite level is calculated the same as (**B**). (**E**) The exhausted subtype estimates for normal and tumor tissue from three cancers with viral etiologies. Samples were grouped as tumor normal (Normal), tumor without viral infection (Tumor Virus−), and tumor with viral infection (Tumor Virus+) in three cancer types: Cervical Squamous Cell Cancer (CESC-HPV), Head and Neck Squamous Cell Cancer (HNSCC-HPV), and Liver Hepatocellular Carcinoma (LIHC). For LIHC samples, both Hepatitis B (HBV) C (HCV) were investigated separately. The following number of normal, tumor (virus−), and tumor (virus+) samples for each cancer type were analyzed: CESC, 3/9/169; HNSCC, 44/241/36; LIHC-HBV, 50/118/44; LIHC-HCV, 50/118/35. For all subfigures, hypotheses were tested using the one-sided Mann–Whitney U test and *p*-values are visualized as follows: ns, *p* ≥ 0.05; *p < 0.05; **p < 0.01; ***p < 0.001. Alternative hypotheses are listed in Supplementary Table 1.

We performed TCSP on a set of previously characterized CD39+ and CD39− sorted cell isolates from Non-small Cell Lung Cancer (NSCLC) and Colorectal Cancer (CRC)^65^. CD39+ tumor infiltrating T cells have been associated with both exhausted and effector memory phenotypes^62,65–67^. As observed previously^66^, both CD39+ and CD39− T cell isolates were estimated to be primarily comprised of the EM subtype (Fig. 3A). In addition, we confirmed that the CD39+ isolate exhibited a higher relative level of exhausted subtype than CD39− T cells (Fig. 3B). These two trends held in both subsets when NSCLC and CRC samples were analyzed individually (Supplementary Fig. 9A and B).

Tumor infiltrating T cells that express a high level of PD-1 have also been associated with exhaustion^12,16,68^. A previously characterized set of T cell isolates from blood and NSCLC tumors^16^ were profiled for TCSs.

Exhausted subtype estimates increased as PD-1 expression increased in isolates, corroborating previous observations (Fig. 3D). EM cells isolated from blood had the lowest exhausted estimates, while PD-1 high isolates from tumors had the highest. Similarly, activated subtype estimates were positively correlated with PD-1 expression. Conversely, naïve and EM subtype estimates were negatively correlated with PD-1 expression (Fig. 3C, Supplementary Fig. 9D). This suggests that expression of PD-1 in CD8+ infiltrates, along with expression of other classical inhibitory receptors (Supplementary Fig. 9C), correlates with a population of cells that are increasingly antigen experienced with a decreasing effector function.

T cell exhaustion and dysfunction may be caused by a variety of factors^12,69^, but are typically associated with persistent, chronic antigen stimulation. This model of exhaustion has its origins in viral research but has also been demonstrated in solid tumors^12^. We investigated viral and tumor induced exhaustion in tandem, by performing TCSP on solid tumors with etiologies involving persistent viral infection. In Cervical Squamous Cell Cancer (CESC)^70^, exhaustion was highest in tumor samples with HPV infection, as previously suggested^71–73^ (Fig. 3E). Interestingly, TCS estimates suggest that naïve T cells decrease in abundance from normal tissue to HPV− to HPV+ tumor tissue (Supplementary Fig. 10A). In Head and Neck Squamous Cell Cancer (HNSCC)^74^, exhausted T cells were most abundant in HPV+ tumor samples, as previously observed^75–78^ (Fig. 3E). In addition, a higher estimated abundance of total T cell infiltrate in HPV+ tumor samples corroborate previous HNSCC research^76,79^ (Supplementary Fig. 10B). In Liver Hepatocellular Cancer (LIHC)^80^, exhaustion trends were mixed. Exhaustion was estimated to be higher in tumors with active HCV infections, but not HBV infections (Fig. 3E). TCS profiles were similar across both malignant and non-malignant tissue and regardless of viral status (Supplementary Fig. 10C and D). Similarly, observations in literature indicate few differences in T cell exhaustion, T cell type, and T cell abundance when comparing viral status in this LIHC dataset^78,80^. Yet, in agreement with our estimates, alternative work has found HCV specific T cells to be highly exhausted^62^. These data suggest that exhausted T cells are elevated in at least some tumor types during concurrent viral infection, but may be dependent on the type of virus. Regardless, TCSP is able to measure heightened levels of exhaustion caused by both viral and tumor induced exhaustion.

### T cell subtype profiling predicts anti-PD-1 response

TCSP is robust in measuring biologically relevant physiology. In addition, T cell biology is heavily implicated in patient response to immunotherapies, especially in HNSCC^13,14^, NSCLC^16,81–84^, and Melanoma^20,85^. Therefore, we used TCSP to study and retrospectively predict anti-PD-1 therapy response in these three cancer types.

**Figure 4:**
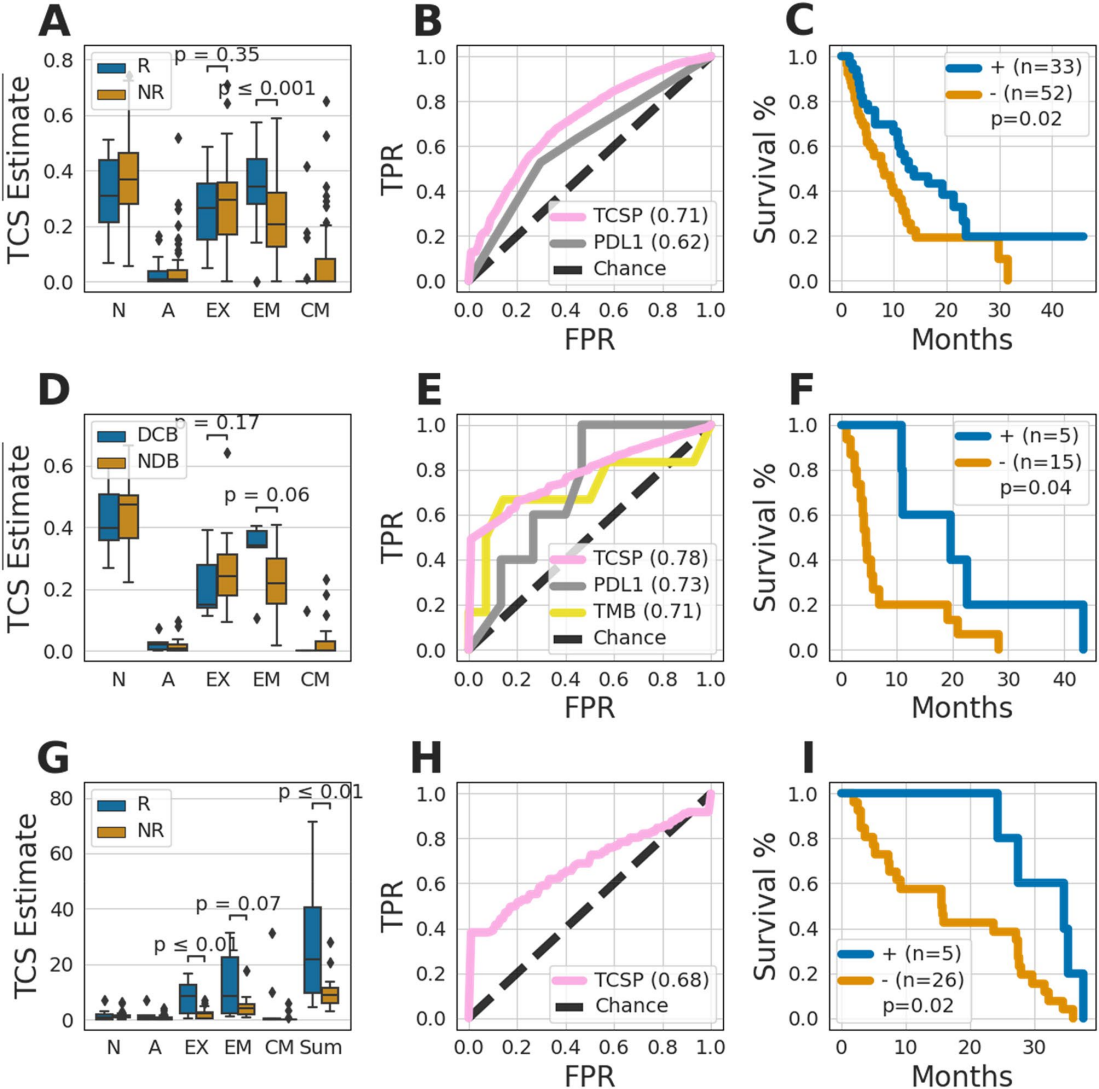
T Cell Subtype Profiling predicts anti-PD1 response. Cohorts of patients with recurrent and metastatic Head and Neck Squamous Cell Cancer (HNSCC, **A**–**C**), recurrent and metastatic Non-small Cell Lung Cancer (NSCLC, **D**–**F**), and recurrent and metastatic Melanoma (**G**–**I**) were explored. Boxplots (**A**,**D**,**G**) show the sum-normalized (**A**,**D**) or non-normalized (**G**) T Cell Subtype estimates grouped by response to anti-PD1 therapy in the respective indications. *R* responder; *NR* non-responder; *DCB* durable clinical benefit; *NDB* non-durable benefit; *N* naïve subtype; *A* activated subtype; *EX* exhausted subtype; *EM* effector memory subtype; *CM* central memory subtype; *Sum* Sum of all five subtypes. The receiver operator characteristic (ROC) curves (**B**,**E**,**H**) of a biomarker derived from TCS estimates (TCSP), and where available, PD-L1 IHC (PDL1), and tumor mutational burden (TMB) are shown for each respective indication. Chance is shown as a dotted black line, and the area under each ROC curve (AUC) is detailed in the legend. Kaplan–Meier plots (**C**,**F**,**I**) of overall survival of patients who were predicted by the TCSP biomarker to be responders (+) and non-responder (−) in each respective indication. The following number of samples were in each cohort: HNSCC (**I**), R = 22 and NR = 63; NSCLC (**D**), DCB = 6 and NDB = 15; Melanoma (**G**), R = 10 and NR = 21. Hypotheses in (**A**,**D**,**G**) were tested using the one-sided Mann–Whitney U test. The alternative hypotheses in (**A**,**D**) are that EM is higher in responders and EX is higher in non-responders. The alternative hypotheses in (**G**) are that EX, EM, and Sum are higher in responders. Hypotheses in (**C**,**F**,**I**) were tested using the log rank test.

First, we examined response in recurrent and metastatic HNSCC. This cohort of 85 samples consists of non-nasopharyngeal samples collected from patients at Washington University School of Medicine and processed at Cofactor Genomics. With TCSP, we found that the EM T cells were more abundant in tumors of responders and that overall T cell infiltrate was higher in responders, in line with other work^13,14^ (Fig. 4A, Supplementary Fig. 11A). Using machine learning, we built a multianalyte biomarker to predict response to anti-PD-1 treatment in this indication. We used bootstrap sampling to best approximate future performance in an independent validation set. Notably, this biomarker (AUC = 0.71) better predicted objective response relative to PD-L1 IHC testing (AUC = 0.62), which is an indicated companion diagnostic in this cohort (Fig. 4B). The TCSP based biomarker also predicted overall survival outcomes, with predicted responders having longer survival (Fig. 4C).

Next, we considered a cohort of recurrent and metastatic NSCLC patients with primary tumors that were treated with anti-PD-1 therapies in early treatment lines^86^. We investigated the differences between 21 patients with durable clinical benefit (DCB) and non-durable benefit (NDB). DCB was defined as complete response (CR), partial response (PR), or stable disease (SD) as defined by RECIST 1.1 for at least 6 months. Similar to HNSCC, we observed a greater proportion of exhausted and EM T cells in NDB and DCB samples, respectively (Fig. 4D, Supplementary Fig. 11B).

These sum-normalized exhausted and EM T cell observations measured from FFPE tissue in two different cancers are reminiscent of the characteristics observed in tumor-isolated T Cells gated on CD39 and PD-1 (Fig. 3A,C) and suggest that CD39+ and/or PD-1+ T cell populations may be higher in responders in both cancers. This corroborates the previously observed association of PD-1+ T cells and response in NSCLC^16^. In this NSCLC cohort, we also observed that estimates of exhaustion were higher in NDB patients, consistent with this previous work (Supplementary Fig. 11B). In contrast with other previous work^81–84^, however, we found that total infiltrate levels were higher in this population of NDB patients (Supplementary Fig. 11B). Similar to HNSCC, we used the TCSP readouts as inputs to train a multianalyte biomarker and evaluate the performance in predicting DCB in NSCLC. The TCSP biomarker better predicted DCB (AUC = 0.78) compared to both the indicated companion diagnostic, PD-L1 IHC (AUC = 0.73), and also Tumor Mutational Burden (AUC = 0.71) (Fig. 4E). In addition, patients predicted to have DCB by the TCSP-based biomarker had significantly longer overall survival (Fig. 4F).

Next, we explored an existing public dataset of advanced Melanoma patients treated with Nivolumab^87^. We investigated the TCSs in 31 on-treatment tumors. Patients who responded to Nivolumab were found to have higher levels of exhausted, EM, and total T cell infiltrate (Fig. 4G), echoing observations in HNSCC (Supplementary Fig. 11A). However, no trends were observed when considering sum-normalized readouts (Supplementary Fig. 11C). These non-normalized observations agreed with some work^85^, while normalized observations failed to corroborate other work^20^, perhaps due to differences in methods of measuring T cell abundance, i.e. IHC vs single cell RNA-seq. In line with the above HNSCC and NSCLC experimentation, we built a third multianalyte biomarker to predict response to Nivolumab. This biomarker also predicted objective response (AUC = 0.69) and overall survival in this third indication (Fig. 4H,I).

Last, we investigated immune gene sets related to inflammation^36^, cytotoxicity^37^, IFNγ^38,39^, antigen presentation^39^, and exhaustion^39^. These gene sets have been associated with response and survival for patients undergoing anti-PD1 therapy in several tumor types including the ones investigate in this work: HNSCC, NSCLC, and melanoma. Using the same multi-analyte and cross validation approach, we evaluated biomarkers using these gene sets in these three cohorts, as compared to TCSP. TCSP performed the best in NSCLC and HNSCC cohorts, only being surpassed in performance by antigen presentation genes in NSCLC (Supplementary Fig. 12). TCSP and antigen presentation genes had the highest average AUC performances across all three cohorts: 0.72 and 0.73, respectively (Supplementary Fig. 12).

Although varying across difference cancers, the TCSP of tumors has shown early promise in predicting clinical outcomes to treatment with anti-PD-1 therapies. Further development considering other additional analytes, especially antigen presentation gene expression, may further improve performance. In addition, additional independent samples are required to validate the biomarkers for clinical use. With its unique ability to characterize FFPE samples, TCSP can facilitate previously impossible translational research. To aid other researchers in characterizing the TCS of their cohorts and discovering other TCSP-based biomarkers in oncology, we have made TCSP available at tcsp.cofactorgenomics.com.

### T cell subtype profiling of many cancers

**Figure 5:**
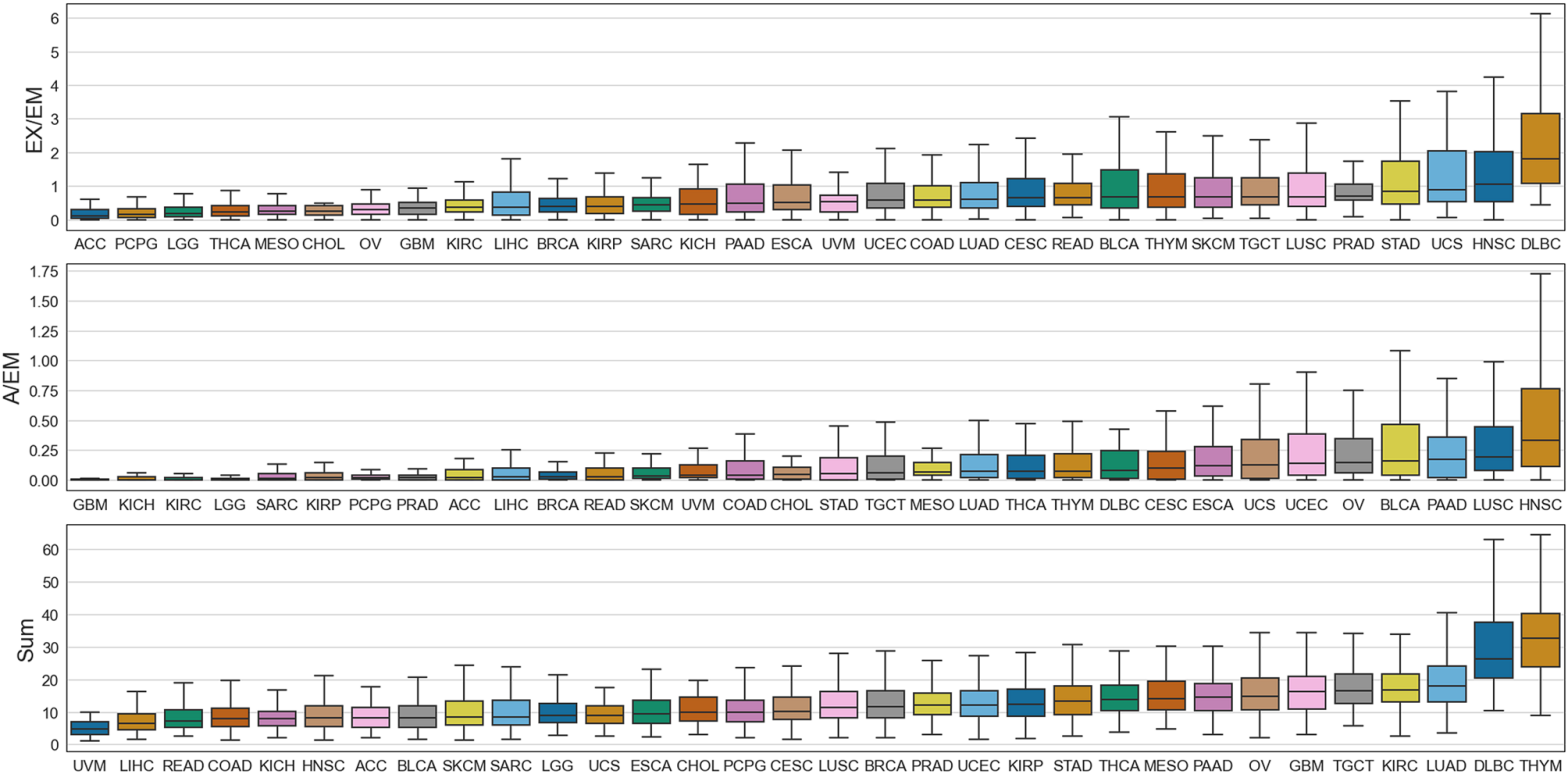
T Cell Subtype Profiling of many cancers. Box and whisker plots show the inter- and intra-tumor variance of various TCGA projects for: the effector memory-normalized exhausted levels (EX/EM), and the effector memory-normalized activated levels (A/EM), and the sum total infiltrate (Sum). Outliers are omitted for visual clarity.

Given the performance of TCSP-based biomarkers in HNSCC, NSCLC, and melanoma, we leveraged TCGA data to expand our investigation to 32 additional indications. We identified additional tumor types in which response to anti-PD-1 might be predicted by searching for tumor types with similar characteristics to HNSCC, NSCLC, and melanoma. The HNSCC, NSCLC, and melanoma cohorts presented in the previous section had a high ratio of exhausted to EM cells (Fig. 4). As expected, in the TCGA data, Head and Neck Squamous Cell Cancer (HNSC), Lung Squamous Cell Cancer (LUSC), and Skin Cutaneous Melanoma (SKCM) were among the eight highest exhausted to EM T cell ratios (Fig. 5). Other indications with a high exhausted to EM ratio include Large B-cell Lymphoma (DLBC), Uterine Carcinosarcoma (UCS), and Stomach adenocarcinoma (STAD). HNSCC and LUSC are the two highest in the ratio of activated to EM, followed by Pancreatic Adenocarcinoma (PAAD), Bladder Urothelial Carcinoma (BLCA), and Ovarian Serous Cystadenocarincoma (OV) (Fig. 5). These additional tumor types are potential candidates for TCSP-based biomarkers to predict anti-PD-1 response.

We also investigated other general immunological trends across tumor types in TCGA. When considering the abundance of many TCSs, the inter-disease variance was as large as intra-disease variance (Supplementary Fig. 13). Several observations fit expectations. Thymoma (THYM) and DLBC had the highest total T cell infiltrate (Fig. 5), while Thyroid Carcinoma (THCA) had the highest presence of the naïve T cells (Supplementary Fig. 13). Almost all cancers lacked CM T cells, except THYM, which had the highest abundance (Supplementary Fig. 13). Other observations may provide new insights. DLBC, Cholangiocarcinoma (CHOL), and UCS were the three highest exhausted diseases (Supplementary Fig. 14). OV had the highest levels of activated T Cells, while Glioblastoma Multiforme (GBM) had the highest abundance of EM T Cells (Supplementary Fig. 13). The TCSP of TCGA cancers discussed here are available for download at the TCSP portal.

## Discussion

The TCSP method described in this paper is a novel way to characterize T cells. Characterizing the five TCSs in FFPE patient samples enables new opportunities for researching the tumor-immune microenvironment, studying response to immunotherapies, and developing biomarkers to predict patient response to treatment. sHEMs were designed to be specific to each TCS, allowing one to discriminate between TCSs in heterogeneous FFPE tumor samples rather than relying on commonly used non-specific markers (e.g., PD-1 as a marker for exhaustion).

Indeed, we found that, with both in vitro and in-patient samples, many traditional markers for T cell exhaustion are correlated with, but not specific to exhaustion. For instance, gene expression of the inhibitory receptors PD-1, TIM3, and LAG3 is increased in activated T cells as well as exhausted T cells during chronic stimulation (Fig. 2A;^49^). In NSCLC patients, PD-1+ CD8+ cells are associated with exhaustion (Fig. 3D;^12,16^), however, these PD-1+ cell isolates are also associated with increased activation (Supplementary Fig. 9D). Likewise, in NSCLC and CRC patients, CD39+ T cells are associated with both exhausted and EM T cells^62,65–67^. While TCSP corroborated these findings, we also found that CD39+ T cells are associated with higher activation, suggesting that CD39 is not a specific marker for exhaustion nor EM T cells (Fig. 3A,B, Supplementary Figs. 8A-B). These findings, paired with observations in literature, suggest a more complex interaction between single-analyte markers and TCSs, and demonstrate that our TCSP method is a more specific way to characterize infiltrating T cells, especially those that are exhausted.

The use of single-analyte surrogates for complex TCSs is likely driven by the difficulty of comprehensively characterizing TCSs, particularly in FFPE tissue. Typically, accurately estimating TCSs requires flow cytometry and/or functional tests using unpreserved tissue. The TCSP method presented here is the first platform for comprehensive and specific profiling of TCSs in FFPE samples, whether in new or existing RNA-seq datasets.

Importantly, TCSP is not only useful for characterization, but also for developing multianalyte biomarkers to predict patient response to immunotherapy. In HNSCC and NSCLC patients, TCSP-based biomarkers outperformed existing biomarkers, namely the companion diagnostic PD-L1 IHC (Fig. 4A–F). Similarly, a TCSP-based biomarker predicted objective response and overall survival in a public melanoma patient cohort (Fig. 4G–I). Additional development and independent cohorts are needed to validate these biomarkers, but this work demonstrates the potential of TCSP for building biomarkers across multiple cancer types. Supporting this idea, we characterized TCSs across 32 cancer types and found potential indications to pursue biomarker development.

Given the utility of TCSP across multiple cancer types, we have created an online portal where other researchers can leverage this technology. At tcsp.cofactorgenomics.com, researchers can perform TCSP on their own samples by uploading RNA-seq counts. Optionally, researchers can upload sample grouping information to compare sets of samples or explore potential TCSP-based biomarkers in their indication of interest. At this portal, researchers can also download the TCSP of TCGA samples for further inquiry into specific diseases. We hope that this TCSP portal may help others discover biomarkers predictive of anti-PD-1 and other therapies.

## Methods

### Isolation of T cell subsets by flow cytometry

Naïve T cells, effector memory T cells, and central memory T cells were isolated by FACS sorting. Cryopreserved human peripheral blood mononuclear cells (PBMCs) from normal healthy donors were obtained from StemExpress (Folsom, CA) and Astarte Biologics (Bothwell, WA). Cryopreserved CD4+ and CD8+ T cells, enriched by negative selection from PBMCs from normal healthy donors, were obtained from StemExpress. Cells were removed from liquid nitrogen storage and rapidly thawed in a 37 °C water bath with gentle hand shaking until only a small piece of ice remained. Cells were transferred to a 50 mL conical centrifuge tube. One mL of prewarmed media (RPMI-1640 (no phenol red) supplemented with 10% FBS, 10 mmol/L HEPES buffer, 1X GlutaMAX, 50 µg/mL gentamicin) was added dropwise to the cells. Fifteen mL prewarmed media was then slowly added. Cells were centrifuged at 200 × *g* for 10 min at room temperature. The supernatant was aspirated, and cells were resuspended in FACS buffer (calcium- and magnesium-free Hank’s balanced salt solution (HBSS) supplemented with 2% FBS). Seventy five µL aliquots of cell suspension (5 million cells) were transferred to tubes containing 25 µl T cell antibody panel (5 µl each of Brilliant Violet 421™ anti-human CD3 (clone UCHT1, BioLegend (San Diego, CA)), PerCP/Cyanine5.5 anti-human CD4 (clone SK3, BioLegend), APC-H7 anti-human CD8 (clone SK1, BD Biosciences (San Jose, CA)), PE anti-human CCR7 (clone G043H7, BioLegend), PE-Cy™7 anti-human CD45RA (clone L48, BD Biosciences), and incubated at 4 °C for 20 min. The cells were washed twice with 1 mL cold FACS buffer by centrifugation at 350 × g, 5 min, 4 °C. Pellets were each resuspended in 100 µL cold FACS buffer and then pooled. SYTOX™ Green dead cell stain (Thermo Fisher, Waltham, MA) was added to a final dilution of 1:1000. FACS sorting was performed using the BD Biosciences Aria Fusion at the Flow Cytometry Research Core Facility at Saint Louis University School of Medicine. Compensation was established using Anti-Mouse Ig, κ/Negative Control Compensation Particles Set (BD Biosciences) for conjugated antibodies, and PBMCs for SYTOX™ Green dead cell stain. Cells were sorted using a 70 µm nozzle into cold sort buffer (80% HBSS, 20% FBS). Gating for T cell subtypes was as follows: naïve CD4+ T cells (CD3+ /CD4+ /CD45RA+ /CCR7+); naïve CD8+ T cells (CD3+ /CD8+ /CD45RA+ /CCR7+); effector memory CD4+ T cells (CD3+ /CD4+ /CD45RA− /CCR7−); effector memory CD8+ T cells (CD3+ /CD8+ /CD45RA− /CCR7−); central memory CD4+ T cells (CD3+ /CD4+ /CD45RA− /CCR7+); central memory CD8+ T cells (CD3+ /CD8+ /CD45RA− /CCR7+); activated CD4+ T Cells (CD3+ /CD4+ /CD45RA+ /CCR7−); activated CD8+ T Cells (CD3+ /CD8+ /CD45RA+ CCR7−). Supplementary ry Figure 15 shows the gating strategy for a representative donor. Sorted lymphocytes were centrifuged at 1000 × *g* for 5 min and pellets lysed in 350 µL Buffer RLT Plus (Qiagen, Germantown, MD) supplemented with 1/100th volume ß-mercaptoethanol. RNA was extracted using the RNeasy Plus Micro Kit (Qiagen, Germantown, MD) according to the manufacturer’s instructions, and used for RNA-seq library preparation and sequencing.

### In vitro T cell exhaustion

The in vitro generation of exhausted T cells was modified from Balkhi, et al.^88^, and performed by STEMCELL Technologies (Vancouver, BC, Canada). Naïve CD8+ T cells were isolated from fresh leukapheresis samples from three normal healthy donors using the EasySep™ Human Naïve CD8+ T Cell Isolation Kit II (STEMCELL Technologies) following the manufacturer’s recommended protocol. The isolated cells were resuspended in media (RPMI supplemented with 10% FBS) to a final concentration of 1.5–2 × 10^6^ cells/mL. One hundred microliter aliquots of cell suspension (1.5–2 × 10^5^ cells) were transferred to 96-well U-bottom plates. Cultures were rested at 37 °C, 5% CO2 for 30 min before the addition of tetrameric antibody complexes (ImmunoCult™ Human CD3/CD28/CD2 T Cell Activator, STEMCELL Technologies). A two-fold working stock of T Cell Activator was first prepared in media at a concentration of 50 µl/ml. One hundred microliters of working stock were then added to wells for a final concentration of 25 µl/ml. Cultures were incubated at 37 °C, 5% CO2 for a total of 14 days with re-stimulation occurring every two days, as follows. Every two days (Day 2, 4, 6, 8, 10 and 12) average viable cell numbers were determined using the Cellometer Auto 2000 Cell Viability Counter (Nexcelom). Cells were pelleted by centrifugation, supernatants were removed and cells washed once with media before being resuspended in 200 μL media containing 25 μL/mL of the T Cell Activator. In each stimulation step, the number of viable cells was readjusted to the same number as originally seeded on Day 0 (1.5–2 × 105 cells/well depending on the donor). Likewise, at each timepoint, triplicate cell pellets were lysed with Buffer RLT Plus (Qiagen, Germantown, MD) supplemented with 1/100th volume ß-mercaptoethanol. RNA was then extracted using the RNeasy Plus Micro Kit (Qiagen) according to the manufacturer’s instructions and used for RNA-seq library preparation and sequencing.

### Cytokine assays

At each 2 day timepoint of the in vitro generation of exhausted T cells, supernatants from triplicate wells were collected and stored at − 80 °C for cytokine evaluation. Cytokine concentrations were measured using the Meso Scale Discovery (MSD®) multiplex immunoassay as follows. On the day of the assay, the V-Plex Custom Human Cytokine Proinflammatory Panel 1 (2-Plex) kit and the supernatant samples were brought to room temperature. The assay plates were washed three times with 150 μL of wash buffer (PBS supplemented with 0.05% Tween-20 (Sigma-Aldrich, Saint Louis, MO). Eight concentrations of the Calibrator Blend (standard) were prepared in Diluent 2 in microcentruge tubes, and 50 μL of each concentration of the Calibrator was added to each assay plate in duplicate. Next, 25 μL of Diluent 2 was added to the remaining wells of the assay plates. Supernatants were diluted 1:100 in PBS supplemented with 1% BSA (Sigma-Aldrich) and 25 μL of each sample (undiluted and 1:100) was added to the assay plates to yield final dilutions of 1:2 and 1:200. The assay plates were sealed with adhesive plate seals and incubated at room temperature on a plate shaker (650 rpm) for 2 h. After the 2 h incubation, the plates were washed three times with 150 μL of wash buffer. The detection antibody solution was prepared by combining 240 μL of each supplied detection antibody (IFNγ and IL2) with 11.52 mL of Diluent 3 and 25 μL of the detection antibody solution was then added to each well. The assay plates were sealed with adhesive plate seals and incubated at room temperature on a plate shaker (650 rpm) for 2 h. After the 2 h incubation, the plates were washed three times with 150 μL of 1 × wash buffer and 150 μL of 2 × Read Buffer T was then added to each well. The plates were read immediately on a Meso QuickPlex SQ 120 Instrument.

### Flow cytometric analysis of T cell exhaustion markers

At each 2 day timepoint of the in vitro generation of exhausted T cells, one sample from each donor was assessed for LAG3, Tim3 and PD-1 expression by flow cytometry. Cells were distributed into a 96-well U-bottom plate for staining. Cells were first washed twice with FACS buffer followed by centrifugation at 1500 rpm for 5 min and removal of the supernatant. Human Fc block (BD Biosciences) was diluted in FACS buffer, and 50 μL of diluted Fc block was then added to each well (1 μg/sample), after which the cells were gently resuspended and incubated for 10 min at room temperature. Antibodies to surface markers CD8, LAG3, Tim3 and PD-1 were used to stain the cells to assess purity by flow cytometry. Working concentrations of the antibodies were prepared in FACS buffer (50 μL per staining point), and 50 μL of each diluted antibody mixture was added to the appropriate wells. For staining controls either previously stimulated and cryopreserved Concanavalin A (ConA) stimulated PBMCs were thawed and used, or extra ImmunoCult™ Human T Cell Activator stimulated cells from the study were used depending on the time point. After the appropriate antibody, or antibody mixture had been added to each well, cells were incubated at 4 °C in the dark for 30 min. At the end of the incubation period, cells were washed twice with FACS buffer, followed by centrifugation at 1500 rpm for 5 min and removal of the supernatant. Cells were then resuspended in 150 μL of FACS buffer followed by addition of the viability dye 7-AAD to the appropriate wells (2 μL/sample). Cells were analyzed by flow cytometry on a Beckman Coulter CytoFLEX Flow Cytometer, collecting 20,000–50,000 cell events (or a maximum of 60 s) per well for each sample. Antibodies were obtained from BioLegend (San Diego, CA): Brilliant Violet 421™ anti-human CD8, FITC anti-human LAG3, PE anti-human TIM3, APC anti-human PD-1.

### RNA-seq library preparation and sequencing

Libraries were prepared using the TruSeq RNA Access Library Prep Kit from Illumina (San Diego, CA) according to the manufacturer’s instructions (naïve, effector memory, central memory); or the NEBNext® Ultra™ II Directional RNA Library Prep Kit for Illumina® (NEB, Ipswich, MA) along with the xGen Exome Research Panel biotinylated oligonucleotide pool and xGen Hybridization and Wash Kit from Integrated DNA Technologies (Coralville, IA) according to the manufacturer’s instructions (naïve, activated, exhausted, HNSCC and NSCLC FFPE specimens). Final libraries were sequenced as single-end 75 base pair reads on a NextSeq500 (Illumina, San Diego, CA) following the manufacturer's protocols.

### Dissociated tumor cells

Cryopreserved dissociated tumor cells from three indications (ovarian adenocarcinoma, lung adenocarcinoma, and melanoma) were obtained from Discovery Life Sciences (Huntsville, AL). Cells were processed and stained for FACS analysis and sorting as described above for cryopreserved PBMCs, except that prior to antibody staining Fc receptors were blocked using Human TruStain FcX™ according to manufacturer’s instructions (BioLegend, San Diego, CA).

### Processing of RNA-seq data

FASTQ files were preprocessed with trim_galore/cutadapt to remove adapter sequences as well as reads with PHRED quality scores less than 20 and reads that were shorter than 20 base pairs. The trimmed reads were aligned to the human genome GRCh38 with STAR using the 2-pass method. Read counts were generated using htseq-counts and annotation from Gencode v22.

### T cell subtype health expression model creation

Differential expression of the five sHEMs was initially performed using DeSeq2. Eight, three, six, five, and three libraries were used for the naive, activated, exhausted, EM, CM subtypes, respectively. EM and CM models were derived from T cell isolates sorted by flow cytometry. Activated and exhausted models were derived from day 4 and days 12 and 14 of the chronic stimulation in vitro experiment, respectively. The naïve model was derived from both T cell isolates sorted by flow cytometry and day 0 cells of the chronic stimulation in vitro experiment. For each subtype, genes were considered in descending order of log fold difference versus all other subtypes. Genes with a coefficient of variation larger than 0.25 and a maximum counts per million (CPM) less than 15 were ignored until 10 genes were chosen. The mean CPM of respective libraries for these selected genes was used to create the preliminary sHEM for each TCS consisting of 123 genes. The genes in these models were then filtered using Cancer Cell Line Encyclopedia (CCLE). Cell lines with disease origins related to immune cells were not considered. Mean expression across all other cell lines was normalized per gene by the max value (CPM) of the five sHEMs. Genes corresponding to a normalized, average CCLE expression ≥ 0.2 were removed from the models. After filtering, the sHEMs were comprised of 46 genes. For analytical validation experiments, certain libraries were omitted to remove bias, e.g. naive, activated, and exhausted libraries from donor 3 were removed to estimate those libraries during the performance evaluation. In addition, for biomarker experiments, sHEMs were optimized for the three indications.

### T cell subtype characterization

Estimation of TCSs can be modelled as a linear combination of the gene expression of each TCS present in the bulk RNA sequencing data: B = S x F, where B is a vector representing the gene expression of the 46 genes from a heterogenous sample comprised of tumor, stroma, and immune cells, S is a 46 by 5 matrix of sHEMs, and F is a vector of length 5 that represents the estimated mRNA fractions of each TCS present in the heterogenous sample. For every sample, S is known, B is sequenced, and T cell Subtype Profiling (TCSP) solves for F. CPMs of each gene of input samples were normalized to the max expression of the sHEMs. Then, linear epsilon Support Vector Regression was used to solve the above equation, yielding estimated mRNA fractions of the TCSs.

### xCell deconvolution

xCell estimations were calculated using the TIMER web service^89^. Of the 39 immune readouts available with xCell (via TIMER), we focused on the 6 that measured T cell states, namely naïve, central memory, and effector memory CD4+ and CD8+ T cells. Although the authors recommend estimating cell abundances with a heterogeneous set of samples, we found that the most plausible results came from estimating T cell isolate samples (CD4+ , CD8+ , N, CM, EM) only, i.e. without FFPE libraries.

### Data creation and access

Exhaustion titrations were created in silico by randomly selecting reads from CD45− libraries of 3 different tumors (Lung, Ovarian, Melanoma) and the day 14 library from donor 3. Mixes with ovarian CD45− libraries were created such that 0, 1, 2, 5, 8, 12, 17, 25, 50, 75, 100% of reads came from the exhausted library, while for mixes with lung and melanoma CD45− libraries, 0, 25, 50, 75, 100% of reads came from the exhausted library. Fastq files for CD39+ isolates (GSE113590)^65^, PD-1+ isolates (GSE99531)^16^, and Melanoma tumor (GSE91061) samples^87^ were downloaded from the European Nucleotide Archive (ENA) via the Aspera transfer tool. Head and Neck Squamous Cell Carcinoma (HNSCC), Cervical Squamous Cell Carcinoma (CESC), and Liver Hepatocellular Carcinoma (LIHC) counts files were downloaded via the GDC/TCGA REST API (https://api.gdc.cancer.gov). For these three datasets, virus status labels were used as published in the respective supplementary materials^70,74,80^.

### Specimens

85 HNSCC samples were collected from pre-immunotherapy tumor tissue obtained from patients with RM-HNSCC that were treated with a PD-1 inhibitor (pembrolizumab or nivolumab). Sequential sections of formalin-fixed and paraffin embedded (FFPE) tissue blocks were utilized for analysis via T Cell Subtype Profiling and the on-label PD-L1 IHC assay. Patients were grouped according to tumor response to immunotherapy using RECIST criteria. The study design was approved by Washington University School of Medicine IRB.

21 NSCLC samples were collected at time of first diagnosis from patients before treatment with a PD-1 inhibitor (pembrolizumab or nivolumab) between April 2013 and January 2018 at the University Hospital Basel, the Cantonal Hospital Baselland, Switzerland, and the St. Clara Hospital Basel. The groups of patients analyzed is a subset of a cohort previously published^86^. PD-L1 IHC and TMB were performed and evaluated as previously described^86^. The study was approved by the local Ethical Review Board (Ethikkommission Nordwestschweiz, Project-ID 2018-01751) and performed in compliance with all relevant ethical regulations.

31 Melanoma samples were a subset of a cohort previously published^87^. Responders are defined as those with complete response and partial response and non-responders are defined as those with progressive disease according to RECIST criteria.

Informed consent was obtained from patients for all specimens according to the local IRBs.

RNA was extracted from HNSCC FFPE samples using the RNAstorm™ Kit (Cell Data Sciences, Fremont, CA). RNA was extracted from NSCLC FFPE samples using the RecoverAll™ Total Nucleic Acid Isolation Kit (Thermo Fisher Scientific, Waltham, MA).

### TCSP-based biomarker creation and analysis

TCSP-based biomarkers were optimized independently for each of the three indications via cross validation. Normalized and/or non-normalized TCSP readouts were used as (5 or 10) input features and were estimated using a single set of sHEMs optimized for all three indications. No other gene expression was included as a feature. Supplementary Table 2 summarizes the sHEMs used for each figure. After feature standardization, several feature projection (Principal Component Analysis, Independent Component Analysis, Kernel Principal Component Analysis) and machine learning algorithms (Adaboost, K-Nearest Neighbors, Random Forest, Support Vector Machine) were evaluated via cross validation. The machine learning (ML) model with the highest cross validated Area Under the Receiver Operating Characteristic curve (AUC) was chosen as the biomarker. Bootstrap sampling was used to cross validate and approximate ML model performance for future independent datasets. In bootstrap sampling, a set of samples are randomly sampled with replacement for training the ML model, with the remaining samples—called the out-of-bag set—used to evaluate the ML model’s performance. This is done iteratively (hundreds of times) and a model’s performance is evaluated by averaging the performance over all out-of-bag samples. Bootstrap sampling is the most rigorous statistical approach to predicting the performance of an ML model in future independent cohorts. Receiver Operating Characteristic (ROC) curves are used to show overall performance of TCSP-based biomarkers in predicting objective response. The curves shown for TCSP-based biomarkers are the mean out-of-bag ROC of the optimal ML model. PD-L1 IHC and TMB ROC curves include all samples without any sampling procedure. Kaplan–Meier plots are used to show the ability for the same TCSP-based biomarkers to predict overall survival. To do so, the average out-of-bag prediction scores of all samples were thresholded at 0.5 to determine if a sample was biomarker positive or negative. All ML model optimization and evaluation was performed with Python (3.8.3) via the Scipy library (1.5.0).

### Gene-set-based biomarker creation and analysis

The genes of six immune-related gene sets (Supplementary Table 3) were used for multi-analyte biomarker creation. The same machine learning algorithms and cross validations approaches used for creating TCSP-based biomarkers were used. In effect, TCSP features were exchanged for gene expression features, while controlling for the same algorithms, e.g. linear PCA+ SVM. A unique model was fit for each of the three tumor types. The mean OOB AUC was calculated for each tumor type, as well as a mean AUC across all tumor types per gene set.

## Supplementary Information

Supplementary Information.

## References

[1] Xin YJ (2020). Trends in clinical development for PD-1/PD-L1 inhibitors. Nat. Rev. Drug Discov..

[2] Vaddepally, R. K., Kharel, P., Pandey, R., Garje, R. & Chandra, A. B. Review of indications of FDA-approved immune checkpoint inhibitors per NCCN guidelines with the level of evidence. *Cancers* **12** (2020). 10.3390/cancers12030738 PMC7140028 32245016

[3] Haslam A, Gill J, Prasad V (2020). Estimation of the percentage of US Patients with cancer who are eligible for immune checkpoint inhibitor drugs. JAMA Netw. Open.

[4] Warner AB (2020). Long-term outcomes and responses to retreatment in patients with melanoma treated with PD-1 blockade. J. Clin. Oncol..

[5] Sheth S (2020). Durvalumab activity in previously treated patients who stopped durvalumab without disease progression. J. Immunother. Cancer.

[6] Havel JJ, Chowell D, Chan TA (2019). The evolving landscape of biomarkers for checkpoint inhibitor immunotherapy. Nat. Rev. Cancer.

[7] Lu S (2019). Comparison of biomarker modalities for predicting response to PD-1/PD-L1 checkpoint blockade: A systematic review and meta-analysis. JAMA Oncol..

[8] Waldman AD, Fritz JM, Lenardo MJ (2020). A guide to cancer immunotherapy: from T cell basic science to clinical practice. Nat. Rev. Immunol..

[9] van den Broek T, Borghans JAM, van Wijk F (2018). The full spectrum of human naive T cells. Nat. Rev. Immunol..

[10] Smith-Garvin JE, Koretzky GA, Jordan MS (2009). T cell activation. Annu. Rev. Immunol..

[11] Mueller SN, Gebhardt T, Carbone FR, Heath WR (2013). Memory t cell subsets, migration patterns, and tissue residence. Annu. Rev. Immunol..

[12] McLane LM, Abdel-Hakeem MS, Wherry EJ (2019). CD8 T cell exhaustion during chronic viral infection and cancer. Annu. Rev. Immunol..

[13] Hanna, G. J. *et al.* Frameshift events predict anti-PD-1/L1 response in head and neck cancer. *JCI Insight* **3**, (2018). 10.1172/jci.insight.98811 PMC5916245 29467336

[14] Hong MH (2019). High CD3 and ICOS and low TIM-3 expression predict favourable survival in resected oesophageal squamous cell carcinoma. Sci. Rep..

[15] Kamphorst AO (2017). Proliferation of PD-1+ CD8 T cells in peripheral blood after PD-1-targeted therapy in lung cancer patients. Proc. Natl. Acad. Sci. USA.

[16] Thommen DS (2018). A transcriptionally and functionally distinct PD-1+ CD8+ T cell pool with predictive potential in non-small-cell lung cancer treated with PD-1 blockade. Nat. Med..

[17] Huang AC (2017). T-cell invigoration to tumour burden ratio associated with anti-PD-1 response. Nature.

[18] Twyman-Saint VC (2015). Radiation and dual checkpoint blockade activate non-redundant immune mechanisms in cancer. Nature.

[19] Im SJ (2016). Defining CD8+ T cells that provide the proliferative burst after PD-1 therapy. Nature.

[20] Sade-Feldman M (2018). Defining T cell states associated with response to checkpoint immunotherapy in melanoma. Cell.

[21] Siddiqui I (2019). Intratumoral Tcf1 + PD-1 + CD8 + T cells with stem-like properties promote tumor control in response to vaccination and checkpoint blockade immunotherapy. Immunity.

[22] Miller BC (2019). Subsets of exhausted CD8+ T cells differentially mediate tumor control and respond to checkpoint blockade. Nat. Immunol..

[23] Terranova-Barberio M (2020). Exhausted T cell signature predicts immunotherapy response in ER-positive breast cancer. Nat. Commun..

[24] Takeuchi Y (2018). Clinical response to PD-1 blockade correlates with a sub-fraction of peripheral central memory CD4+ T cells in patients with malignant melanoma. Int. Immunol..

[25] Schillebeeckx, I. *et al.* Analytical performance of an immunoprofiling assay based on RNA models. *J. Mol. Diagn.* **22**, (2020). 10.1016/j.jmoldx.2020.01.009 32036085

[26] Paik S (2004). A multigene assay to predict recurrence of tamoxifen-treated, node-negative breast cancer. N. Engl. J. Med..

[27] Hao Y (2019). Analytical verification performance of Afirma genomic sequencing classifier in the diagnosis of cytologically indeterminate thyroid nodules. Front. Endocrinol. (Lausanne).

[28] Drukker CA (2013). A prospective evaluation of a breast cancer prognosis signature in the observational RASTER study. Int. J. Cancer.

[29] Yoshihara, K. *et al.* Inferring tumour purity and stromal and immune cell admixture from expression data. *Nat. Commun.* **4**, (2013). 10.1038/ncomms3612 PMC3826632 24113773

[30] Newman AM (2015). Robust enumeration of cell subsets from tissue expression profiles. Nat. Methods.

[31] Steen CB, Liu CL, Alizadeh AA, Newman AM (2020). Profiling cell type abundance and expression in bulk tissues with CIBERSORTx. Methods Mol. Biol..

[32] Finotello F (2019). Molecular and pharmacological modulators of the tumor immune contexture revealed by deconvolution of RNA-seq data. Genome Med..

[33] Aran D, Hu Z, Butte AJ (2017). xCell: Digitally portraying the tissue cellular heterogeneity landscape. Genome Biol..

[34] Becht E (2016). Estimating the population abundance of tissue-infiltrating immune and stromal cell populations using gene expression. Genome Biol..

[35] Racle J, Gfeller D (2020). EPIC: A tool to estimate the proportions of different cell types from bulk gene expression data. Methods Mol. Biol..

[36] Sangro B (2020). Association of inflammatory biomarkers with clinical outcomes in nivolumab-treated patients with advanced hepatocellular carcinoma. J. Hepatol..

[37] Danilova L (2016). Association of PD-1/PD-L axis expression with cytolytic activity, mutational load, and prognosis in melanoma and other solid tumors. Proc. Natl. Acad. Sci. USA.

[38] Chow, L. Q. M. *et al.* Biomarkers and response to pembrolizumab (pembro) in recurrent/metastatic head and neck squamous cell carcinoma (R/M HNSCC). **34**, 6010–6010 (2016). 10.1200/JCO.2016.34.15_suppl.6010.

[39] Ayers M (2017). IFN-γ–related mRNA profile predicts clinical response to PD-1 blockade. J. Clin. Investig..

[40] Barretina J (2012). The cancer cell line encyclopedia enables predictive modelling of anticancer drug sensitivity. Nature.

[41] Wu, G. & Haw, R. Functional interaction network construction and analysis for disease discovery. in *Methods in Molecular Biology* vol. 1558 235–253 (Humana Press Inc., 2017). 10.1007/978-1-4939-6783-4_11 28150241

[42] Willinger T (2006). Human naive CD8 T cells down-regulate expression of the WNT pathway transcription factors lymphoid enhancer binding Factor 1 and transcription Factor 7 (T cell Factor-1) following antigen encounter in vitro and in vivo. J. Immunol..

[43] Sekiya, T. *et al.* The nuclear orphan receptor Nr4a2 induces Foxp3 and regulates differentiation of CD4+ T cells. *Nat. Commun.* **2**, (2011). 10.1038/ncomms1272 PMC3104557 21468021

[44] Hardie DL (2011). The stromal cell antigen CD248 (endosialin) is expressed on naive CD8+ human T cells and regulates proliferation. Immunology.

[45] Ding, T. *et al.* DUSP8 phosphatase: Structure, functions, expression regulation and the role in human diseases. *Cell Biosci.* **9** (2019). 10.1186/s13578-019-0329-4 PMC6712826 31467668

[46] Carl JW, Bai X-F (2008). IL27: Its roles in the induction and inhibition of inflammation. Int. J. Clin. Exp. Pathol..

[47] Malek TR (2008). The biology of interleukin-2. Annu. Rev. Immunol..

[48] Frucht, D. M. IL-23: A cytokine that acts on memory T cells. *Sci. Signal.* **2002**, pe1–pe1 (2002). 10.1126/stke.2002.114.pe1 11784889

[49] Kanhere A (2012). T-bet and GATA3 orchestrate Th1 and Th2 differentiation through lineage-specific targeting of distal regulatory elements. Nat. Commun..

[50] Huard B, Tournier M, Hercend T, Triebel F, Faure F (1994). Lymphocyte-activation gene 3/major histocompatibility complex class II interaction modulates the antigenic response of CD4+ T lymphocytes. Eur. J. Immunol..

[51] Anderson AC, Joller N, Kuchroo VK (2016). Lag-3, Tim-3, and TIGIT: Co-inhibitory receptors with specialized functions in immune regulation. Immunity.

[52] Li Y, Ohms SJ, Sun C, Fan J (2013). NF-κB controls Il2 and Csf2 expression during T cell development and activation process. Mol. Biol. Rep..

[53] Heuzé ML (2005). ASB2 is an elongin BC-interacting protein that can assemble with cullin 5 and Rbx1 to reconstitute an E3 ubiquitin ligase complex. J. Biol. Chem..

[54] Bakos E (2017). CCR2 regulates the immune response by modulating the interconversion and function of effector and regulatory T cells. J. Immunol..

[55] Gunturi A, Berg RE, Forman J (2004). The role of CD94/NKG2 in innate and adaptive immunity. Immunol. Res..

[56] Imbratta C, Hussein H, Andris F, Verdeil G (2020). c-MAF, a Swiss army knife for tolerance in lymphocytes. Front. Immunol..

[57] Contento RL (2008). CXCR4-CCR5: A couple modulating T cell functions. Proc. Natl. Acad. Sci. USA.

[58] Sintes J (2013). Cutting edge: Ly9 (CD229), a SLAM family receptor, negatively regulates the development of thymic innate memory-like CD8 + T and invariant NKT cells. J. Immunol..

[59] Nolz JC, Harty JT (2014). IL-15 regulates memory CD8+ T cell O-glycan synthesis and affects trafficking. J. Clin. Invest..

[60] Mann TH, Kaech SM (2019). Tick-TOX, it’s time for T cell exhaustion. Nat. Immunol..

[61] Wherry EJ (2007). Molecular signature of CD8+ T cell exhaustion during chronic viral infection. Immunity.

[62] Gupta PK (2015). CD39 expression identifies terminally exhausted CD8+ T cells. PLOS Pathog..

[63] Sommermeyer D (2016). Chimeric antigen receptor-modified T cells derived from defined CD8+ and CD4+ subsets confer superior antitumor reactivity in vivo. Leukemia.

[64] Taylor GS, Long HM, Brooks JM, Rickinson AB, Hislop AD (2015). The immunology of epstein-barr virus-induced disease. Annu. Rev. Immunol..

[65] Simoni Y (2018). Bystander CD8+ T cells are abundant and phenotypically distinct in human tumour infiltrates. Nature.

[66] Canale FP (2018). CD39 expression defines cell exhaustion in tumor-infiltrating CD8+ T cells. Cancer Res..

[67] Duhen T (2018). Co-expression of CD39 and CD103 identifies tumor-reactive CD8 T cells in human solid tumors. Nat. Commun..

[68] Ma, J. *et al.* PD1Hi CD8+ T cells correlate with exhausted signature and poor clinical outcome in hepatocellular carcinoma. *J. Immunother. Cancer* **7**, (2019). 10.1186/s40425-019-0814-7 PMC6884778 31783783

[69] Thommen DS, Schumacher TN (2018). T cell dysfunction in cancer. Cancer Cell.

[70] Burk RD (2017). Integrated genomic and molecular characterization of cervical cancer. Nature.

[71] Meng Y (2018). PD-L1 expression correlates with tumor infiltrating lymphocytes and response to neoadjuvant chemotherapy in cervical cancer. J. Cancer.

[72] Liu C (2017). Increased expression of PD-L1 by the human papillomavirus 16 E7 oncoprotein inhibits anticancer immunity. Mol. Med. Rep..

[73] Papasavvas E (2016). High-risk oncogenic HPV genotype infection associates with increased immune activation and T cell exhaustion in ART-suppressed HIV-1-infected women. Oncoimmunology.

[74] Lawrence MS (2015). Comprehensive genomic characterization of head and neck squamous cell carcinomas. Nature.

[75] Gameiro, S. F. *et al.* Treatment-naïve HPV+ head and neck cancers display a T-cell-inflamed phenotype distinct from their HPV- counterparts that has implications for immunotherapy. *Oncoimmunology* **7**, (2018). 10.1080/2162402X.2018.1498439 PMC6169583 30288365

[76] Lechner A (2017). Characterization of tumor-associated T-lymphocyte subsets and immune checkpoint molecules in head and neck squamous cell carcinoma. Oncotarget.

[77] Krishna S (2018). Human papilloma virus specific immunogenicity and dysfunction of CD8+ T cells in head and neck cancer. Cancer Res..

[78] Cao, S. *et al.* Dynamic host immune response in virus-associated cancers. *Commun. Biol.* **2**, (2019). 10.1038/s42003-019-0352-3 PMC6430765 30911684

[79] Mandal R (2016). The head and neck cancer immune landscape and its immunotherapeutic implications. JCI Insight.

[80] Ally A (2017). Comprehensive and integrative genomic characterization of hepatocellular carcinoma. Cell.

[81] Hu-Lieskovan S (2019). Tumor characteristics associated with benefit from pembrolizumab in advanced non–small cell lung cancer. Clin. Cancer Res..

[82] Garon EB (2015). Pembrolizumab for the treatment of non–small-cell lung cancer. N. Engl. J. Med..

[83] Sun R (2018). A radiomics approach to assess tumour-infiltrating CD8 cells and response to anti-PD-1 or anti-PD-L1 immunotherapy: An imaging biomarker, retrospective multicohort study. Lancet Oncol..

[84] Teng MWL, Ngiow SF, Ribas A, Smyth MJ (2015). Classifying cancers basedon T-cell infiltration and PD-L1. Can. Res..

[85] Tumeh PC (2014). PD-1 blockade induces responses by inhibiting adaptive immune resistance. Nature.

[86] Alborelli I (2020). Tumor mutational burden assessed by targeted NGS predicts clinical benefit from immune checkpoint inhibitors in non-small cell lung cancer. J. Pathol..

[87] Riaz N (2017). Tumor and microenvironment evolution during immunotherapy with nivolumab. Cell.

[88] Balkhi MY, Wittmann G, Xiong F, Junghans RP (2018). YY1 upregulates checkpoint receptors and downregulates type I cytokines in exhausted Chronically Stimulated Human T Cells. iScience.

[89] Li T (2017). TIMER: A web server for comprehensive analysis of tumor-infiltrating immune cells. Cancer Res..

