# Supplementary material for: T cell subtype profiling measures exhaustion and predicts anti-PD-1 response
Source: Sci Rep. 2022 Jan 25;12:1342. doi: 10.1038/s41598-022-05474-7 (PMC8789795; doi:10.1038/s41598-022-05474-7)
Supplement: Supplementary file 1 — Supplementary Information. [file 41598_2022_5474_MOESM1_ESM.docx]

# Supplement

## Supplementary Figure 1


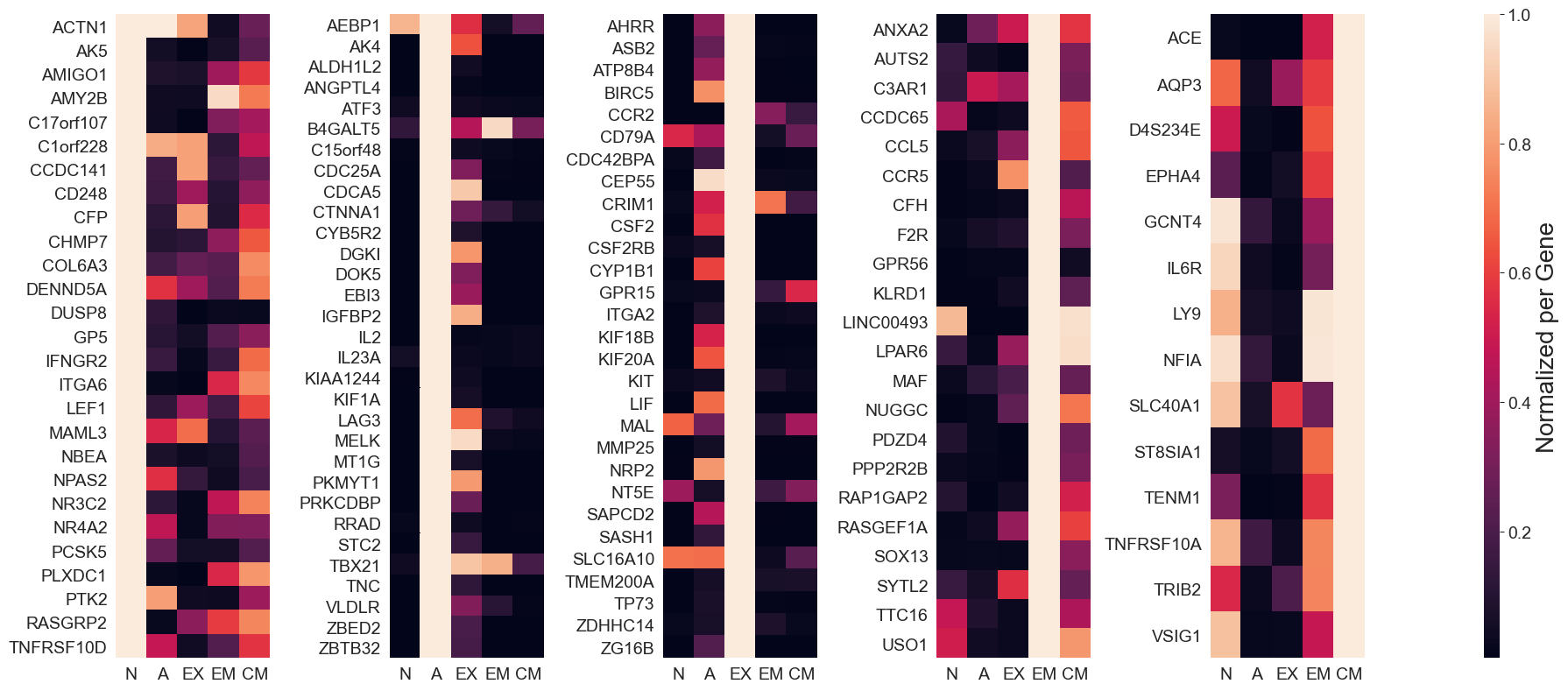


**Supplementary Figure 1: Subtype Health Expression Models before CCLE filtering.** A heatmap shows the gene-normalized expression of genes comprising the 5 T Cell Subtype Health Expression Models (sHEMs) before CCLE filtering. The genes are split up across 5 columns for visual ease. N, naïve model; A, activated model; EX, exhausted model; EM, effector memory model; CM, central memory model.

## Supplementary Figure 2


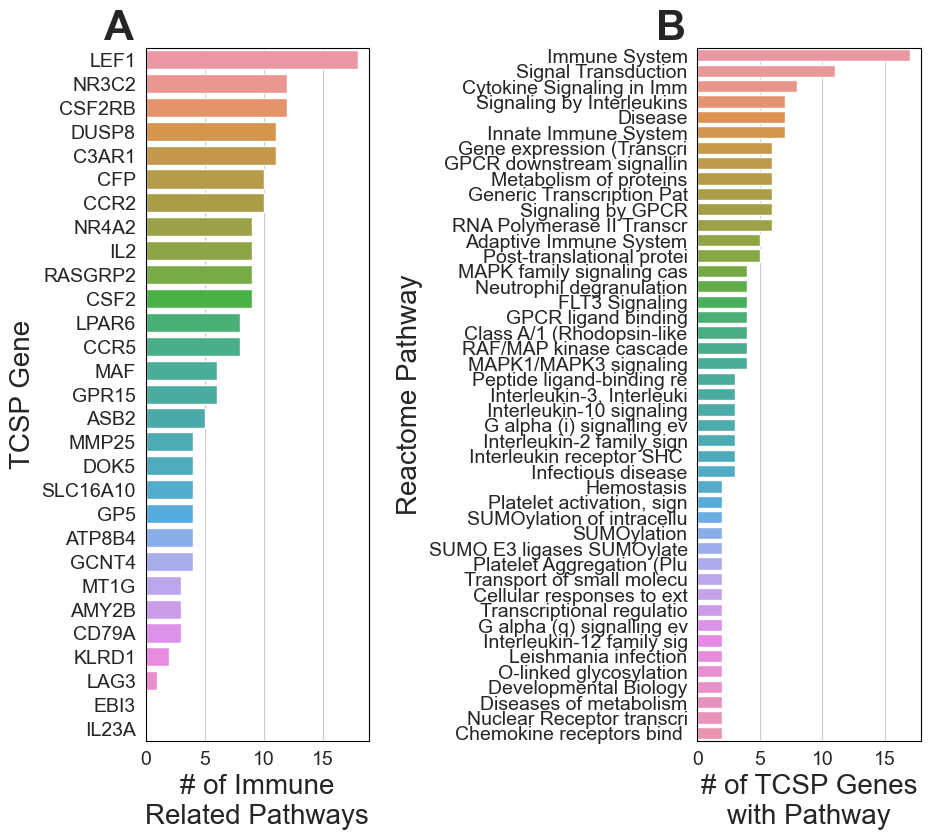


**Supplementary Figure 2: Reactome pathways associated with component genes of Subtype Health Expression Models.** A) Using the Reactome Database, the number of immune related pathways were counted for each gene. Immune related pathways included 'immune', 'infect', 'inflammatory', 'interleukin', 'platelet', 'tcell', 'hemo', 'hiv', 'lymph', 'clot', 'myeloid', 'cytokine', and 'chemokine'. B) The frequency of individual pathways found across all subtypes.

## Supplementary Figure 3


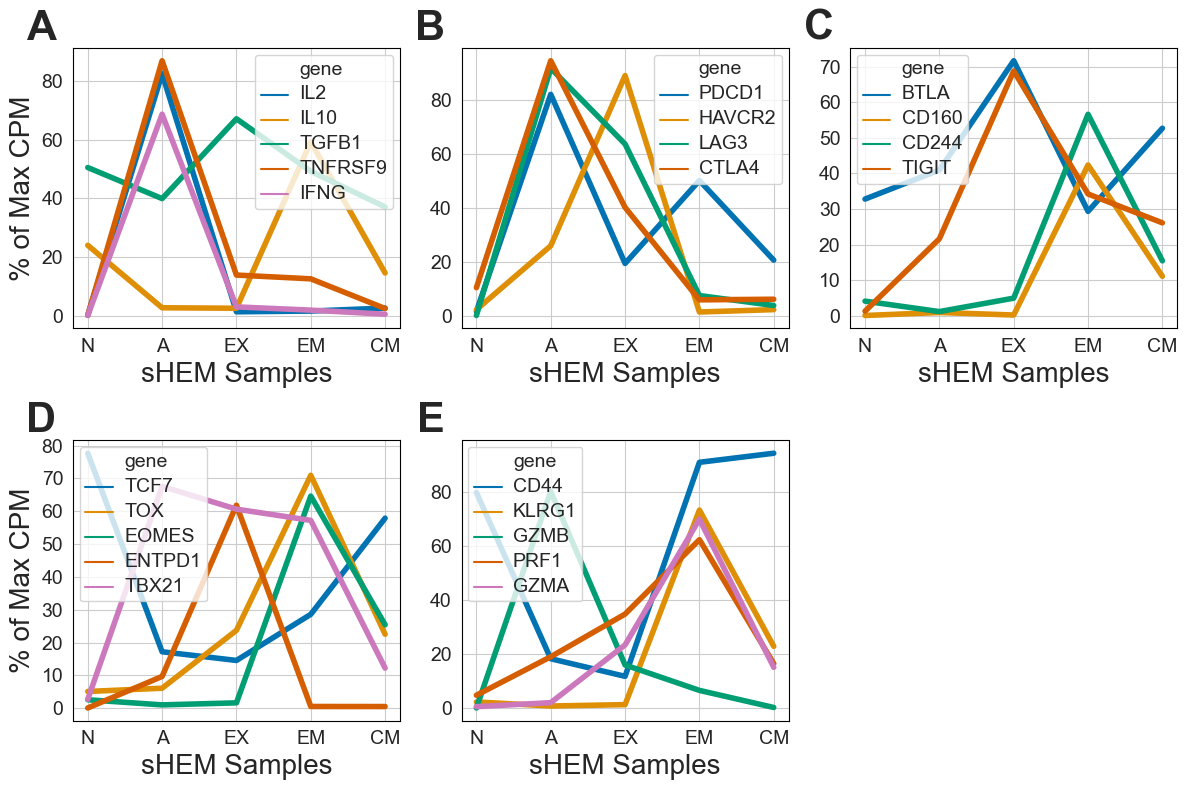


**Supplementary Figure 3: Gene expression of Subtype Health Expression Models.** Average expression across all donors for each Subtype Health Expression Model (sHEM) is calculated as Counts per Million (CPM). Expression is normalized across sHEMs for each gene. Expression is shown for A) activation-associated cytokines, B) exhaustion-associated inhibitory receptors, C) other inhibitory receptors, D) exhaustion associated transcription factors, E) effector associated genes. N, naïve model; A, activated model; EX, exhausted model; EM, effector memory model; CM, central memory model.

## Supplementary Figure 4


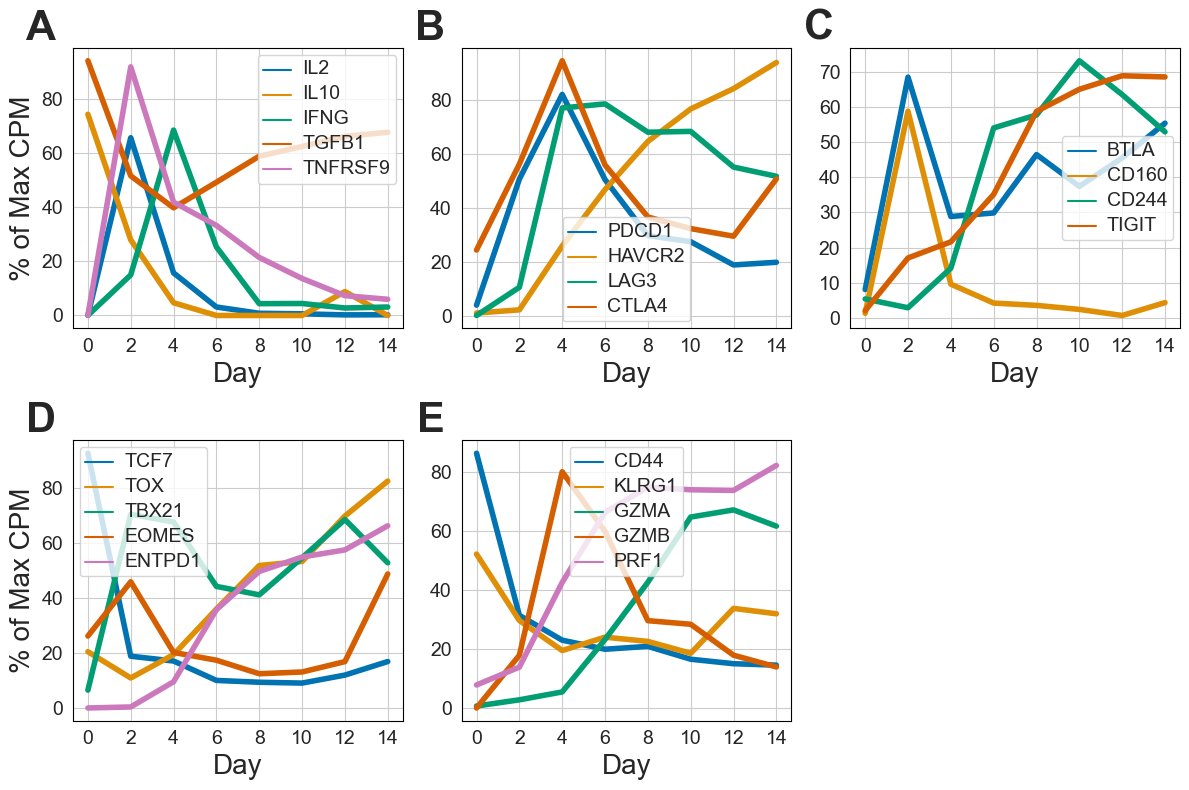


**Supplementary Figure 4: Gene expression of CD8+ T Cells during chronic stimulation *in vitro*.** Average gene expression across donors (n=3) is calculated in Counts per Million (CPM). Expression is normalized across time points for each gene. Expression is shown for A) activation-associated cytokines, B) exhaustion-associated inhibitory receptors, C) other inhibitory receptors, D) exhaustion-associated transcription factors, E) effector-associated genes.

## Supplementary Figure 5


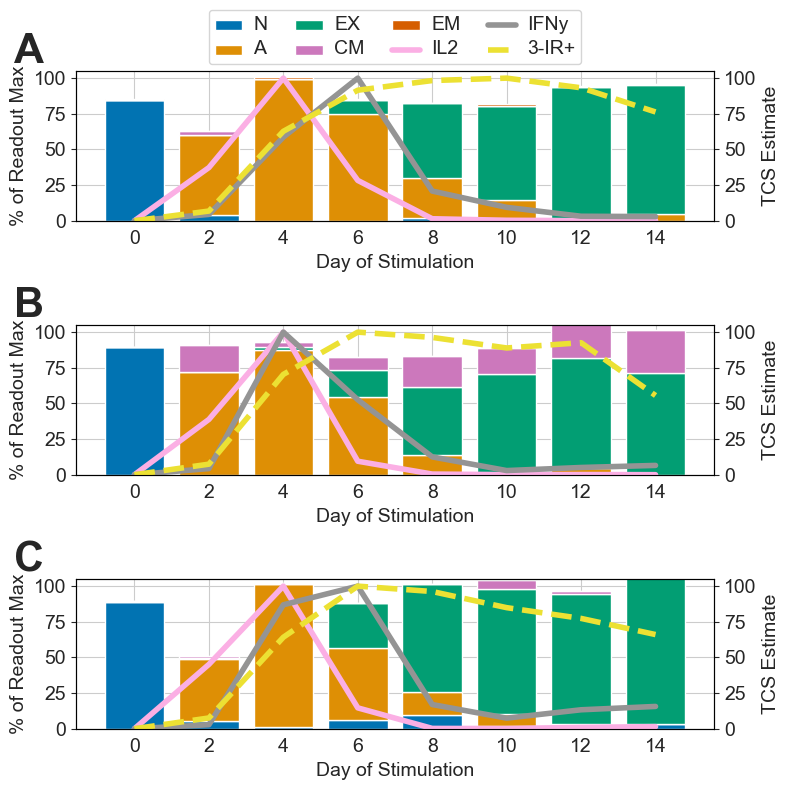


**Supplementary Figure 5: sHEMs evaluation of a chronic stimulation *in vitro model* with different withheld donors.** The same samples and data as Figure 2A were considered. Naïve CD8+ T cells were isolated from three donors and stimulated for 14 days. Donor 3, 2, or 1 was withheld for evaluation when creating the Subtype Health Expression Models (sHEMs) for A (same as Figure 2A), B, and C, respectively. Every two days, the presence of the extracellular cytokines IL2 and IFNγ was measured via ELISA and the abundance of cells jointly expressing the PD1, TIM3, and LAG3 inhibitory receptors (3-IR+) was measured via flow cytometry. These readouts are normalized to their max expression during the time series and shown as lines (left y-axis). Cells were harvested every two days and characterized using T Cell Subtype Profiling (TCSP). T Cell subtype (TCS) estimates are shown as stacked bars (right y-axis). N, naïve subtype; A, activated subtype; EX, exhausted subtype; EM, effector memory subtype; CM, central memory subtype.

## Supplementary Figure 6


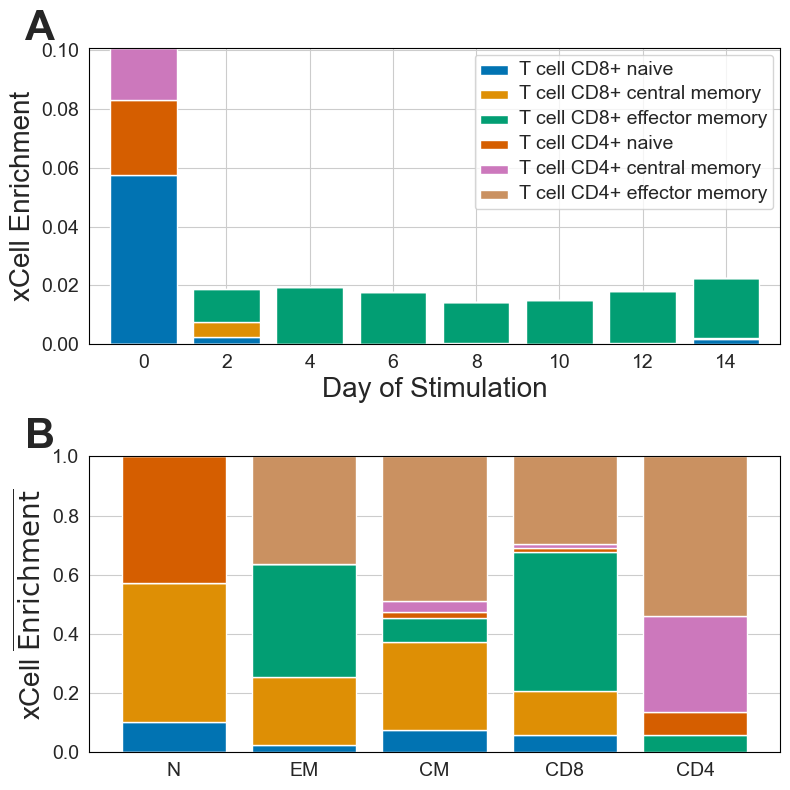


**Supplementary Figure 6: xCell T Cell State Enrichment.** A) The transient change of naïve CD8+ T cells during a chronic stimulation *in vitro* model presented in Figure 2A. xCell enrichment is presented as a stacked barplot (y-axis) for 6 states (naïve, central memory, and effector memory, each for both CD4+ and CD8+) for each timepoint of chronic stimulation (x-axis). B) The sum-normalized xCell enrichment scores (xCell $\overline{\text{Estimate}}$) of T Cell populations isolated from blood (presented in Figure 2B): naïve CD8+ T cells (N, n=5), effector memory CD8+ T Cells (EM, n=5), central memory CD8+ T Cells (CM, n=3), CD8+ T cells (CD8, n=8), and CD4+ T cells (CD4, n=8).

## Supplementary Figure 7


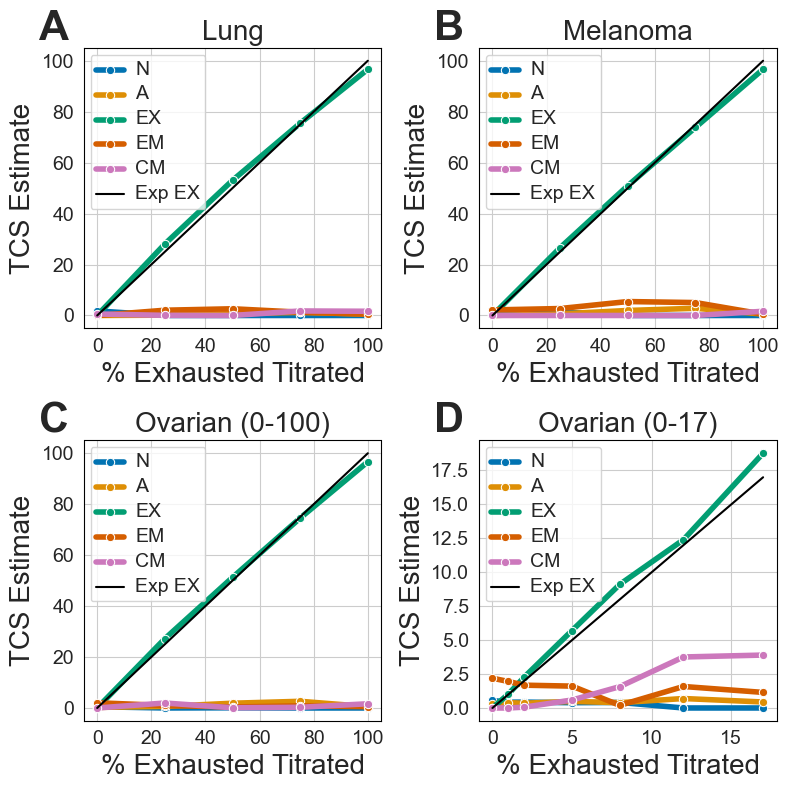


**Supplementary Figure 7: T Cell Subtype Profiling of progressively exhausted samples.** These samples are comprised of varying fractions of chronically stimulated CD8+ T cells (day 14 from Figure 3A) and CD45- cells isolated from tumor samples (from Figure 3C). The expected exhausted subtype estimates (Exp EX) are shown as a black line. Different CD45- isolates were mixed and shown as follows: A), Lung Adenocarcinoma (Lung); B), Melanoma; C) and D), Ovarian Adenocarcinoma (Ovarian). D) is the same data as C), but zoomed in on lower titrations of chronical stimulated CD8+ T Cells. N, naïve subtype; A, activated subtype; EX, exhausted subtype; EM, effector memory subtype; CM, central memory subtype.

## Supplementary Figure 8

**
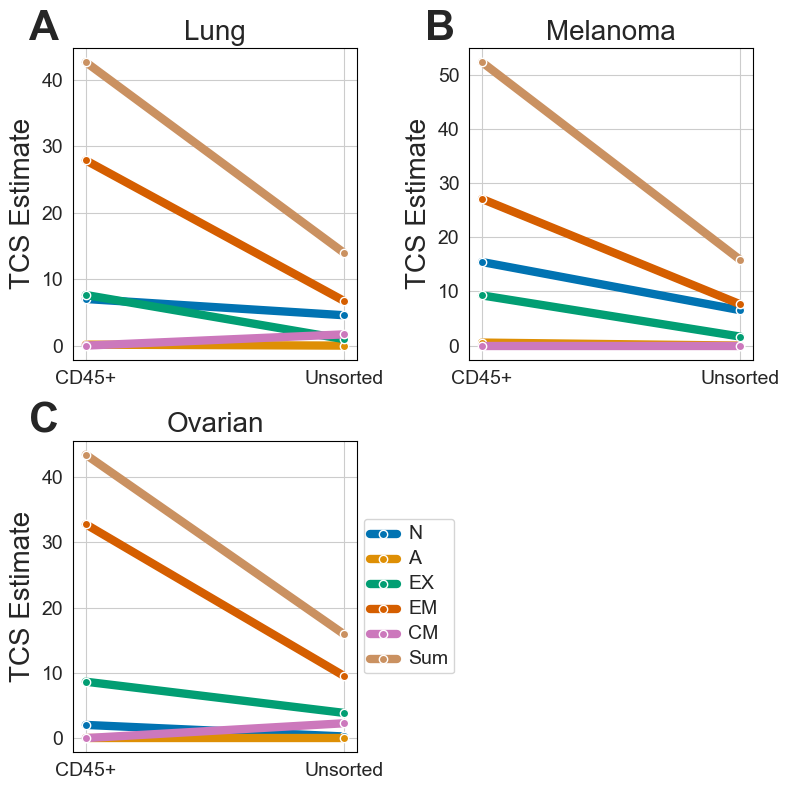
**

**Supplementary Figure 8: T Cell Subtype Profiling of tumor samples.** Cells from fresh tumors (Unsorted) were dissociated and sorted for CD45+ immune isolates (CD45+). Estimates are shown for three different tumor types (from Figure 3C: A), Lung Adenocarcinoma (Lung); B), Melanoma; and C), Ovarian Adenocarcinoma (Ovarian). N, naïve subtype; A, activated subtype; EX, exhausted subtype; EM, effector memory subtype; CM, central memory subtype; Sum, Sum of all five subtypes.

## Supplementary Table 1

| **Figure** | **Random Variable** | **Alternative Hypothesis** |
| --- | --- | --- |
| 3A | A | CD39- < CD39+ |
|  | EX | CD39- < CD39+ |
|  | EM | CD39- > CD39+ |
| 3B | EX/EM | CD39- < CD39+ |
| 3C | A | EM-B < PD1h |
|  |  | PD1n < PD1h |
|  |  | PD1i < PD1h |
|  | EX | EM-B < PD1h |
|  |  | PD1n < PD1h |
|  |  | PD1i < PD1h |
|  | EM | EM-B > PD1h |
|  |  | PD1n > PD1h |
|  |  | PD1i > PD1h |
| 3D | EX/EM | EM-B < PD1h |
|  |  | PD1n < PD1h |
|  |  | PD1i < PD1h |
| 3E | EX - CESC | Normal < Virus+ |
|  |  | Normal < Virus- |
|  |  | Virus- < Virus+ |
|  | EX - HNSCC | Normal < Virus+ |
|  |  | Normal < Virus- |
|  |  | Virus- < Virus+ |
|  | EX – LIHC (HBV) | Normal < Virus+ |
|  |  | Normal < Virus- |
|  |  | Virus- < Virus+ |
|  | EX – CESC (HCV) | Normal < Virus+ |
|  |  | Normal < Virus- |
|  |  | Virus- < Virus+ |

**Supplementary Table 1: Alternative hypotheses for Figure 3.**

## Supplementary Figure 9


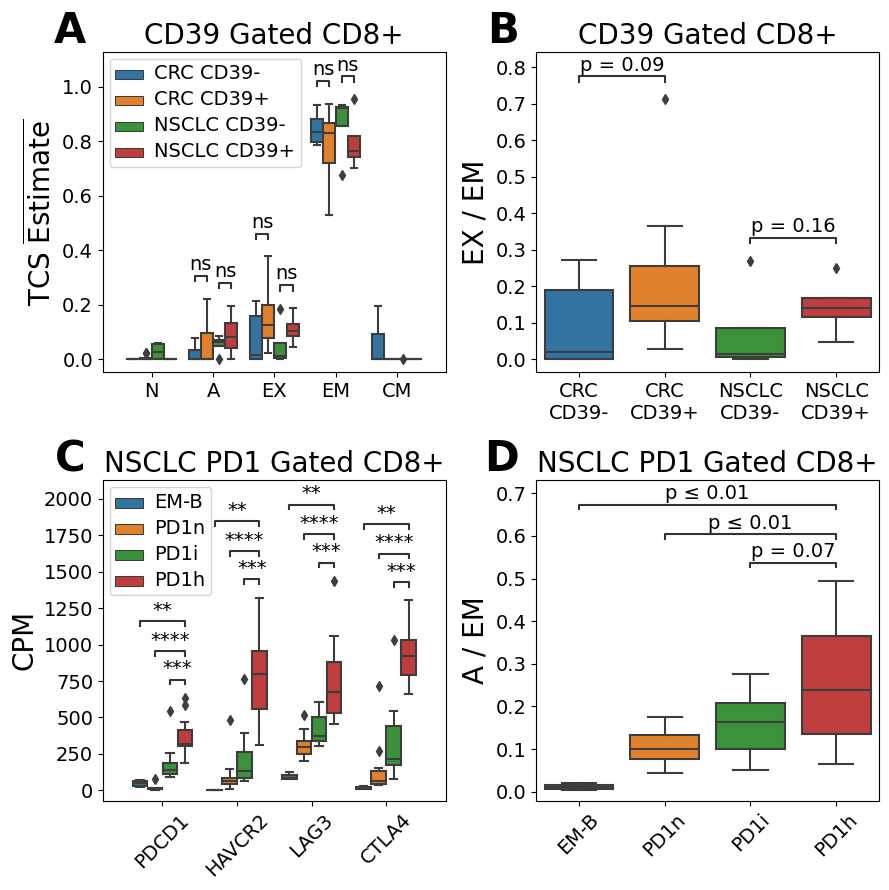


**Supplementary Figure 9: CD39 and PD1 extended data.** A) The sum-normalized T Cell subtype (TCS) estimates (TCS $\overline{\text{Estimate}}$) for CD39- and CD39+ CD8+ T cells isolated from Non-small Cell Lung Cancer (NSCLC, n=4 / m=4) and Colorectal Cancer (CRC, n=7 / m=8) tumors. In both NSCLC and CRC, the alternative hypothesis is that activated and exhausted subtype estimates are higher in CD39+ isolates, while the EM subtype estimate is lower in CD39+ isolates. N, naïve subtype; A, activated subtype; EX, exhausted subtype; EM, effector memory subtype; CM, central memory subtype. B) A composite exhaustion level for CD39- and CD39+ isolates from A. The composite level is calculated as exhausted divided by EM. In both NSCLC and CRC, the alternative hypothesis is that the composite exhaustion level is larger in CD39+ isolates. C) The gene expression, in CPM, of exhaustion associated inhibitory receptors for CD8+ T cell isolates sorted by varying levels of PD1 expression measured by flow cytometry. EM CD8+ T cells isolated from blood (EM-B, n=4) were compared against CD8+ T cells isolated from NSCLC tumors with no PD1 (PD1n, n=11), intermediate PD1 (PD1i, n=11), and high PD1 (PD1h, n=11) expression. For each gene, expression of PD1h isolates are hypothesized to be larger than EM-B, PD1n, and PD1i, respectively. D) A composite activation level for PD1 isolates from C. The composite level is calculated as activated divided by EM. The alternative hypothesis is that the composite activation level is larger in PD1h isolates than EM-B, PD1n, and PD1i, respectively. For all subfigures, hypotheses were tested using the one-sided Mann-Whitney U test and p-values are denoted or visualized as follows: ns, p>=0.05; *, p<0.05; **, p<0.01; ***, p<0.001; ****, p<0.0001.

## Supplementary Figure 10


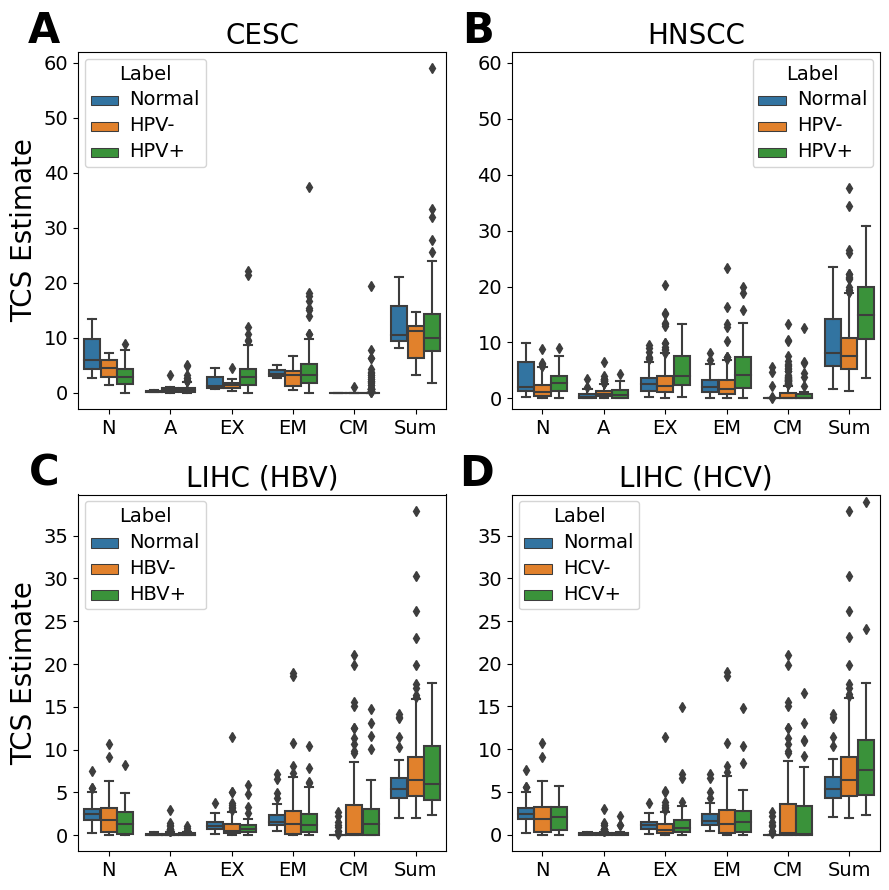


**Supplementary Figure 10: T Cell Subtype Profiling of normal and tumor tissue from three cancers with viral etiologies.** Samples were grouped as tumor normal (Normal), tumor without viral infection (Tumor Virus-), and tumor with viral infection (Tumor Virus+) in three cancer types: Cervical Squamous Cell Cancer (CESC, A), Head and Neck Squamous Cell Cancer (HNSCC, B), and Liver Hepatocellular Carcinoma (LIHC, C and D). For LIHC samples, both Hepatitis B (HBV, C) and Hepatitis C (HCV, D) were investigated separately. There are the following normal, tumor (virus-), and tumor (virus+) samples for each cancer type: CESC, 3/9/169; HNSCC, 44/241/36; LIHC-HBV, 50/118/44; LIHC-HCV, 50/118/35. N, naïve subtype; A, activated subtype; EX, exhausted subtype; EM, effector memory subtype; CM, central memory subtype; Sum, Sum of all five subtypes.

## Supplementary Figure 11


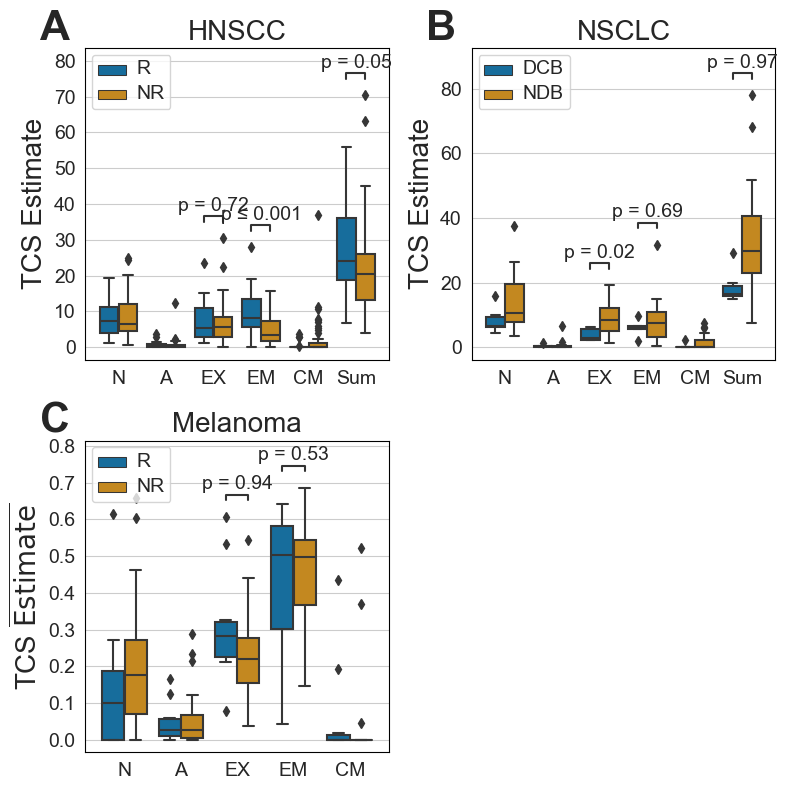


**Supplementary Figure 11: T Cell Subtype Profiling of responders and non-responders to anti-PD1 in three different indications.** A) The T Cell Subtype Profiling (TCSP) of a cohort of patients with recurrent and metastatic Head and Neck Squamous Cell Cancer (HNSCC). N, naïve subtype; A, activated subtype; EX, exhausted subtype; EM, effector memory subtype; CM, central memory subtype; Sum, Sum of all five subtypes. The alternative hypothesis is that EX, EM, and Sum are higher in responders (R, n=22) vs non-responders (NR, n=63). B) The TCSP of a cohort of patients with recurrent and metastatic Non-small Cell Lung Cancer (NSCLC). The alternative hypothesis is that EM, and Sum are higher, while EX is lower in patients with durable clinical benefit (DCB, n=6) vs non-durable benefit (NDB, n=15). C) The sum-normalized TCSP (TCS $\overline{\text{Estimate}}$) of a cohort of patients with recurrent and metastatic Melanoma. The alternative hypothesis is that EX is lower and EM is higher in responders (R, n=10) vs non-responders (NR, n=21). Hypotheses were tested using the one-sided Mann-Whitney U test.

## Supplementary Figure 12

**Supplementary Figure 12: Immune-related gene set biomarker performance versus TCSP biomarker.** The area under the receiver operator characteristic curve (AUC) is shown per gene set for cohorts of patients with recurrent and metastatic Head and Neck Squamous Cell Cancer (HNSCC), recurrent and metastatic Non-small Cell Lung Cancer (NSCLC), and recurrent and metastatic Melanoma (Melanoma). The mean AUC per each gene set across all three cohorts (Average). The gene sets, their constituent genes, and references are detailed in Supplementary Table 3.

## Supplementary Figure 13


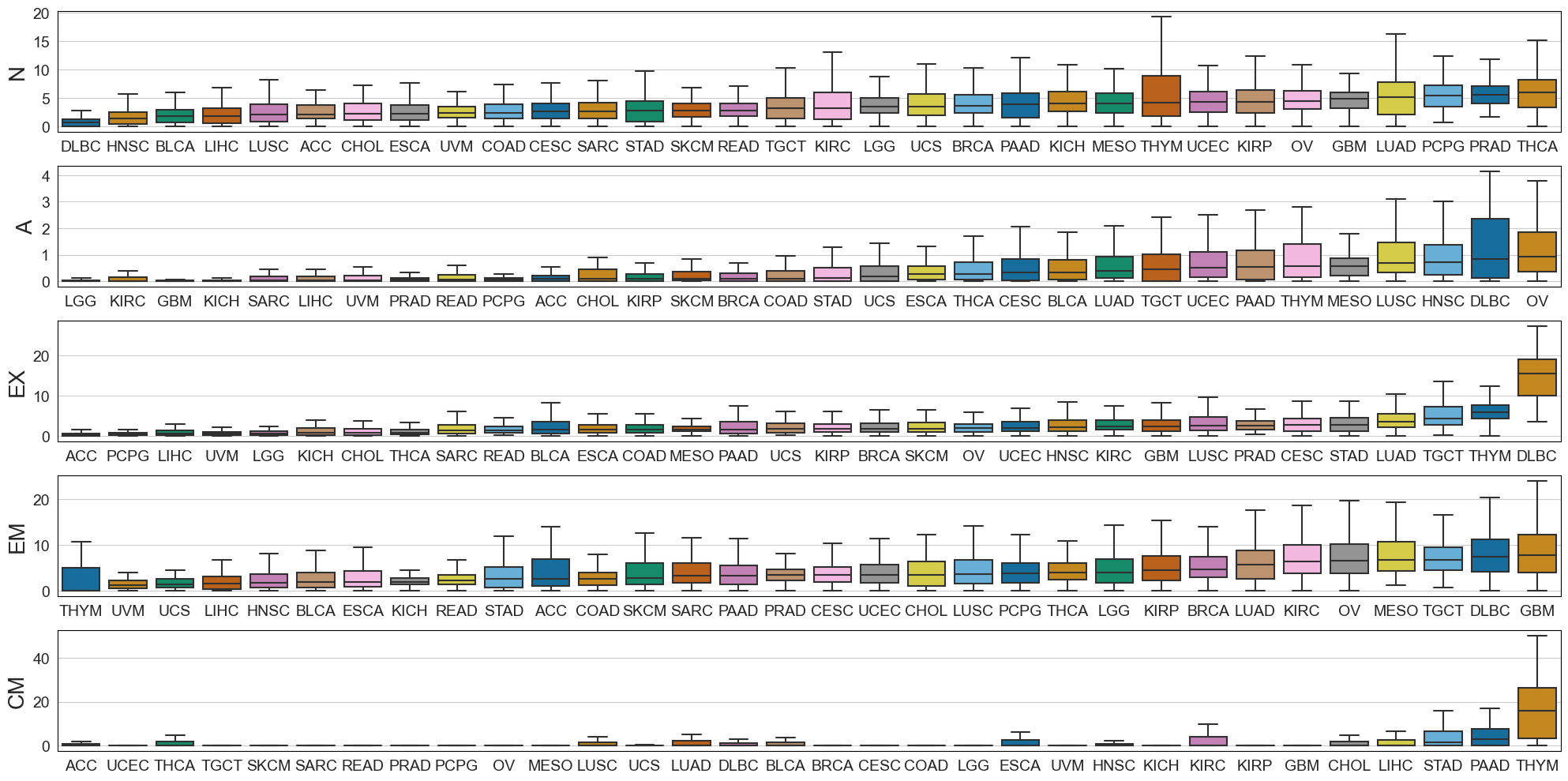


**Supplementary Figure 13: T Cell Subtype Profiling of many cancers.** Box and whisker plots show the inter- and intra-tumor variance of various TCGA projects for the levels of naïve (N), activated (A), exhausted (EX), effector memory (EM), and central memory (CM) T Cell subtypes. Outliers are omitted for visual clarity.

## Supplementary Figure 14


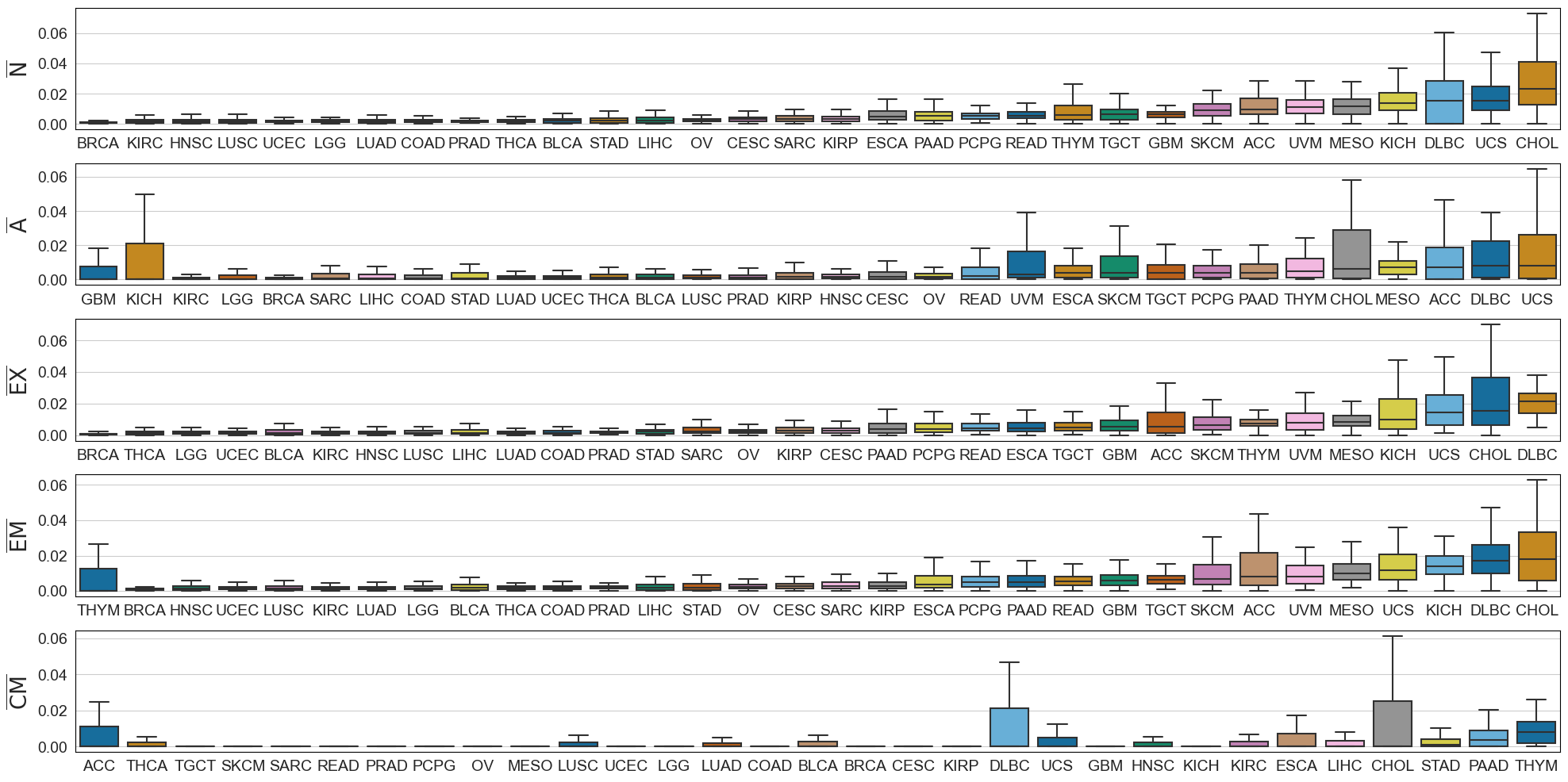


**Supplementary Figure 14: Normalized T Cell Subtype Profiling of many cancers.** Box and whisker plots show the inter- and intra-tumor variance of various TCGA projects for the levels of sum-normalized T Cell subtypes: naïve (N), activated (A), exhausted (EX), effector memory (EM), and central memory (CM). Outliers are omitted for visual clarity.

## Supplementary Figure 15

**Supplementary Figure 15: Gating strategy of naïve, effector memory (EM), central memory (CM), and activated (“Eff”) T cell isolates for a representative donor.**

## Supplementary Table 2

| **Figure** | **sHEMs** | **Left out Donors** | **Rationale** |
| --- | --- | --- | --- |
| 1, 2B-E, 3, 5  Sup 1-4, 6-9, 11-12 | 46 gene | All donors used. | Maximum data used. |
| 2A  Sup 5A | 50 gene | Donor 3 (N, A, Ex)  Donor A5705 (N, EM, CM) | Donor left out for sHEM unbiased evaluation. |
| Sup 5B | 52 gene | Donor 2 (N, A, Ex)  Donor A5705 (N, EM, CM) | Donor left out for sHEM unbiased evaluation. |
| Sup 5C | 50 gene | Donor 1 (N, A, Ex)  Donor A5705 (N, EM, CM) | Donor left out for sHEM unbiased evaluation. |
| 4  Sup 10 | 49 gene | Donor 3 (N, A, Ex)  Donor A5737 (N, EM, CM) | Optimized for anti-PD-1 prediction. |

**Supplementary Table 2: sHEMs used throughout work.**

## Supplementary Table 3

| **Gene Signature** | **Genes** | **Reference** |
| --- | --- | --- |
| Inflammation | *CD274 (PD-L1), CD8A, LAG3, STAT1* | Sangro 2020 ^36^ |
| Cytotoxicity | *GZMA, PRF1* | Danilova 2016 ^37^ |
| IFNγ − 6 gene | *IDO1, CXCL10, CXCL8, CLA-DRA, STAT1, IFNG* | Chow 2016 ^38^, Ayers 2017 ^39^ |
| IFNγ − 18 gene | *IDO1, CXCL10, CXCL9, HLA-DRA, STAT1, IFNG, CD3D, IDO1, CIITA, CD3E, CCL5, GZMK, CD2, HLA-DRA, CXCL13, IL2RG, NKG7, HLA-E, CXCR6, LAG3, TAGAP, CXCL10, STAT1, GZMB* | Ayers 2017 ^39^ |
| Antigen Presentation | *CMKLR1, HLA-DQA1, HLA-DRB1, PSMB10* | Ayers 2017 ^39^ |
| Exhaustion | *CD274 (PD-L1), CD276, CD8A, LAG3, PDCD1LG2, TIGIT* | Ayers 2017 ^39^ |

**Supplementary Table 3: Immune related gene sets considered.**
